# Supplementary material for: Electronic Floquet gyro-liquid crystal
Source: Nat Commun. 2021 Sep 6;12:5299. doi: 10.1038/s41467-021-25511-9 (PMC8421454; doi:10.1038/s41467-021-25511-9)
Supplement: Supplementary file 1 — Supplementary information [file 41467_2021_25511_MOESM1_ESM.pdf]

Supplementary Information
Electronic Floquet Gyro-Liquid Crystal

Iliya Esin,[1,2,*] Gaurav Kumar Gupta,[1] Erez Berg,[3] Mark S. Rudner,[4] and Netanel H. Lindner[1]

[1] *Physics Department, Technion, 3200003 Haifa, Israel*
[2] *Department of Physics, California Institute of Technology, Pasadena, CA 91125, USA*
[3] *Department of Condensed Matter Physics, Weizmann Institute of Science, Rehovot, 76100, Israel*
[4] *Center for Quantum Devices and Niels Bohr International Academy, Niels Bohr Institute, University of Copenhagen, 2100 Copenhagen, Denmark*

This supplemental material provides detailed analytical derivations discussed in the paper and explains key aspects of the numerical simulations. Throughout the notes we work in natural units in which $\hbar = k_B = 1$.

Supplementary Note 1. EFFECTIVE TWO-BAND FLOQUET HAMILTONIAN

Here we derive an effective time-independent Hamiltonian in the rotating wave approximation (RWA), $H_{\rm RWA}(\boldsymbol{k})$ for the periodically driven system described by Supplementary Eq. (1) in the main text, without the interaction part. Our goal is to find the effective dispersion and eigenstates near the resonance ring. The time-dependent Hamiltonian is given by $H(\boldsymbol{k},t) = H_0(\boldsymbol{k}) + H_{\rm d}(t)$, where $H_0(\boldsymbol{k}) = E_0 + \boldsymbol{d}(\boldsymbol{k})\cdot\boldsymbol{\sigma}$, with

$$\boldsymbol{d}(\boldsymbol{k}) = \left(\lambda_0 k_x, \lambda_0 k_y, \frac{|\boldsymbol{k}|^2}{2m_*} + \frac{E_{\rm g}}{2}\right), \tag{1}$$

and $H_{\rm d}(t) = \boldsymbol{v}\cdot\boldsymbol{\sigma}\cos(\Omega t)$, where $\boldsymbol{v} = (0,0,V)$. In the RWA, we shift the lower (upper) bands by $\pm\Omega/2$ and then neglect rapidly oscillating terms. The operator that shifts the energies reads

$$\mathcal{U}_{\rm R}(\boldsymbol{k},t) = e^{\frac{i\Omega t}{2}\hat{\boldsymbol{d}}(\boldsymbol{k})\cdot\boldsymbol{\sigma}}, \tag{2}$$

where $\hat{\boldsymbol{d}}(\boldsymbol{k})$ is a unit vector in the direction $\boldsymbol{d}(\boldsymbol{k})$. The transformed Hamiltonian, $H_{\rm R}(\boldsymbol{k},t)$, then reads

$$H_{\rm R}(\boldsymbol{k},t) = \mathcal{U}_{\rm R}(\boldsymbol{k},t)H(\boldsymbol{k},\boldsymbol{t})\mathcal{U}_{\rm R}^\dagger(\boldsymbol{k},t) - i\mathcal{U}_{\rm R}\frac{\partial\mathcal{U}_{\rm R}^\dagger}{\partial t}. \tag{3}$$

Note that $\mathcal{U}_{\rm R}$ commutes with H_0, but does not commute with the time-derivative and the driving field. Using the explicit form of $H(\boldsymbol{k},t)$, we arrive at

$$H_{\rm R} = E_0 + (|\boldsymbol{d}| - \Omega/2)\hat{\boldsymbol{d}}\cdot\boldsymbol{\sigma} + \boldsymbol{v}'(t)\cdot\boldsymbol{\sigma}\cos(\Omega t), \tag{4}$$

where $\boldsymbol{v}'(t) = \boldsymbol{v}\cos(\Omega t) - \hat{\boldsymbol{d}}\times\boldsymbol{v}\sin(\Omega t) + \hat{\boldsymbol{d}}(\hat{\boldsymbol{d}}\cdot\boldsymbol{v})(1-\cos(\Omega t))$, following from the Rodrigues' formula of rotation of $\boldsymbol{v}$ around $\hat{\boldsymbol{d}}$. The last term in Supplementary Eq. (4) has constant in time parts and parts oscillating with the frequency Ω and 2Ω. Following RWA, we omit the time-oscillating terms, leading to

$$H_{\rm RWA}(\boldsymbol{k}) = E_0 + \boldsymbol{d}_{\rm F}(\boldsymbol{k})\cdot\boldsymbol{\sigma}, \tag{5}$$

where $\boldsymbol{d}_{\rm F} = (|\boldsymbol{d}| - \Omega/2)\hat{\boldsymbol{d}} + \frac{1}{2}[\boldsymbol{v} - \hat{\boldsymbol{d}}(\hat{\boldsymbol{d}}\cdot\boldsymbol{v})]$. The spectrum of $H_{\rm RWA}$ then reads, $\varepsilon_{\boldsymbol{k}\pm} = E_0 \pm \varepsilon_{\boldsymbol{k}}$, where

$$\varepsilon_{\boldsymbol{k}} = \sqrt{(|\boldsymbol{d}| - \Omega/2)^2 + |\boldsymbol{v} - \hat{\boldsymbol{d}}(\hat{\boldsymbol{d}}\cdot\boldsymbol{v})|^2/4}. \tag{6}$$

For simplicity of notations, throughout we set $E_0 = 0$. To leading order in V/Ω and $\lambda_0^2 m/E_{\rm g}$, the minimum of the upper band is positioned at the resonance ring, given by $|\boldsymbol{d}(\boldsymbol{k})| = \Omega/2$. The radius of the resonance ring is given by $k_{\rm R} = \sqrt{m_*\delta E}$ and the quasienergy gap at $|\boldsymbol{k}| = k_{\rm R}$ reads $\Delta_{\rm F} = 2V\lambda_0 k_{\rm R}/\Omega$. We thus approximate the quasienergy as

$$\varepsilon_{\boldsymbol{k}} \approx \sqrt{\left(\frac{k^2}{2m_*} - \frac{\delta E}{2}\right)^2 + \left(\frac{\Delta_{\rm F} k}{2k_{\rm R}}\right)^2}, \tag{7}$$

where $k \equiv |\boldsymbol{k}|$.

The eigenstates of $H_{\rm RWA}$ read

$$|\phi'_{\boldsymbol{k}\pm}\rangle = \frac{1}{\sqrt{2}}\left(\sqrt{1 \pm \hat{\boldsymbol{z}}\cdot\hat{\boldsymbol{d}}_{\rm F}}|\uparrow\rangle \mp e^{i\theta_{\boldsymbol{k}}}\sqrt{1 \mp \hat{\boldsymbol{z}}\cdot\hat{\boldsymbol{d}}_{\rm F}}|\downarrow\rangle\right). \tag{8}$$

where $\theta_{\boldsymbol{k}} = \tan^{-1}(k_y/k_x)$ and $\hat{\boldsymbol{d}}_{\rm F}$ is a unit vector in the direction of $\boldsymbol{d}_{\rm F}$, defined in Supplementary Eq. (5).

At the resonance ring, $\hat{\boldsymbol{d}}_{\rm F}$ approximately lies in the $x-y$ plane such that $\hat{\boldsymbol{z}}\cdot\hat{\boldsymbol{d}}_{\rm F} \approx 0$, therefore

$$|\phi'_{\boldsymbol{k}\pm}\rangle\Big|_{|\boldsymbol{k}|=k_{\rm R}} = \frac{1}{\sqrt{2}}(|\uparrow\rangle \mp e^{i\theta_{\boldsymbol{k}}}|\downarrow\rangle). \tag{9}$$

To find the time-dependent eigenstates of $H(\boldsymbol{k},t)$ within the RWA in the lab frame, we apply the inverse transformation, $\mathcal{U}_{\rm R}^\dagger$ on the states, $|\phi'_{\boldsymbol{k}\pm}\rangle$. Approximating, $\mathcal{U}_{\rm R}^\dagger \approx e^{-\frac{i\Omega t}{2}\sigma^z}$, we arrive at

$$|\phi_{\boldsymbol{k}\pm}(t)\rangle\Big|_{|\boldsymbol{k}|=k_{\rm R}} = \frac{1}{\sqrt{2}}(e^{-i\Omega t}|\uparrow\rangle \mp e^{i\theta_{\boldsymbol{k}}}|\downarrow\rangle). \tag{10}$$

Supplementary Note 2. DERIVATION OF $U_{\rm c}$

In this section we solve the mean-field equation [Eq. (2) in the main text] in the limit $\boldsymbol{h}(t) \to 0$. The solution in this limit provides the minimal interaction strength required for non-zero magnetization, $U_{\rm c}$ [Eq. (10) in the

main text]. In our analysis we assume contact interactions, $\mathcal{V}_{\boldsymbol{k}} = U/\varpi$, and take only the first harmonic of the circularly polarized magnetization field,

$$\boldsymbol{h}(t) = h_1 e^{i\Omega t}(\hat{\boldsymbol{x}} - i\hat{\boldsymbol{y}})/2 + c.c., \tag{11}$$

as discussed in the main text [see Eq. (8)], where h_1 is real and positive. In the rotating frame of reference such a term reads $\mathcal{U}_{\mathrm{R}}(\boldsymbol{h}(t)\cdot\boldsymbol{\sigma})\mathcal{U}_{\mathrm{R}}^{\dagger} = h_1\sigma^x$.

We first rewrite Eq. (2) in the main text in terms of the single particle states and the distribution function, $f_{\boldsymbol{k}\nu}$,

$$\boldsymbol{h}(t) = -U\sum_{\nu=\pm}\int \frac{d^2\boldsymbol{k}}{(2\pi)^2}\langle\phi_{\boldsymbol{k}\nu}(t)|\boldsymbol{\sigma}|\phi_{\boldsymbol{k}\nu}(t)\rangle f_{\boldsymbol{k}\nu}. \tag{12}$$

We approximate $|\phi_{\boldsymbol{k}\nu}(t)\rangle$ by eigenstates of the rotating-wave approximated mean-field Hamiltonian [Eq. (3) in the main text] in the lab frame, given by $H_{\mathrm{MF,RWA}}(\boldsymbol{k},t) = \mathcal{U}_{\mathrm{R}}^{\dagger}H'_{\mathrm{MF,RWA}}(\boldsymbol{k})\mathcal{U}_{\mathrm{R}}$, where

$$H'_{\mathrm{MF,RWA}}(\boldsymbol{k}) = H_{\mathrm{RWA}}(\boldsymbol{k}) + h_1\sigma^x; \tag{13}$$

$H_{\mathrm{RWA}}(\boldsymbol{k})$ is given in Supplementary Eq. (5).

Next, we transform Supplementary Eq. (12) given in a vector form, into a scalar equation for a single mode amplitude, h_1, defined in Supplementary Eq. (11). To this end, we transform the Floquet states into a rotating frame of reference, leading to

$$\boldsymbol{h}(t) = -U\sum_{\nu=\pm}\int \frac{d^2\boldsymbol{k}}{(2\pi)^2}\langle\phi'_{\boldsymbol{k}\nu}|\mathcal{U}_{\mathrm{R}}\boldsymbol{\sigma}\mathcal{U}_{\mathrm{R}}^{\dagger}|\phi'_{\boldsymbol{k}\nu}\rangle f_{\boldsymbol{k}\nu}. \tag{14}$$

Here $|\phi'_{\boldsymbol{k}\nu}\rangle \equiv \mathcal{U}_{\mathrm{R}}(\boldsymbol{k},t)|\phi_{\boldsymbol{k}\nu}(t)\rangle$ are the eigenstates of $H'_{\mathrm{MF,RWA}}(\boldsymbol{k})$, given in Supplementary Eq. (13). Using the Rodrigues' formula, we find, $\mathcal{U}_{\mathrm{R}}(\boldsymbol{k},t)\boldsymbol{\sigma}\mathcal{U}_{\mathrm{R}}^{\dagger}(\boldsymbol{k},t) = \boldsymbol{\sigma}\cos(\Omega t) + \hat{\boldsymbol{d}}\times\boldsymbol{\sigma}\sin(\Omega t) + \hat{\boldsymbol{d}}(\hat{\boldsymbol{d}}\cdot\boldsymbol{\sigma})(1-\cos(\Omega t))$. We extract only the components proportional to $e^{i\Omega t}$, and multiply both sides of Supplementary Eq. (14) by $(\hat{\boldsymbol{x}} + i\hat{\boldsymbol{y}})/\sqrt{2}$. Approximating $\hat{\boldsymbol{d}} \approx \hat{\boldsymbol{z}}$, we find

$$\frac{h_1}{\sqrt{2}} = -U\sum_{\nu=\pm}\int \frac{d^2\boldsymbol{k}}{(2\pi)^2}\langle\phi'_{\boldsymbol{k}\nu}|\frac{\sigma^x + i\sigma^y}{\sqrt{2}}|\phi'_{\boldsymbol{k}\nu}\rangle f_{\boldsymbol{k}\nu}, \tag{15}$$

where $f_{\boldsymbol{k}\nu}$ is the particle distribution function. As $H'_{\mathrm{MF,RWA}}(\boldsymbol{k})$ is symmetric to reflections of the y-axis, the expectation value of σ^y vanishes, leading to

$$h_1 = -U\sum_{\nu=\pm}\int \frac{d^2\boldsymbol{k}}{(2\pi)^2}\langle\phi'_{\boldsymbol{k}\nu}|\sigma^x|\phi'_{\boldsymbol{k}\nu}\rangle f_{\boldsymbol{k}\nu}. \tag{16}$$

To evaluate the integral in Supplementary Eq. (16), in what follows we assume a low-excitation steady-state corresponding to almost-full lower Floquet band and low density of electron and hole excitations in the bottom of the upper and top of the lower Floquet bands, respectively. We then split the integral into two contributions, $h_1 = h_1^{\mathrm{fb}} + h_1^{\mathrm{ex}}$. Here, h_1^{fb} is the contribution of the full lower Floquet band,

$$h_1^{\mathrm{fb}} = -U\int \frac{d^2\boldsymbol{k}}{(2\pi)^2}\langle\phi'_{\boldsymbol{k}-}|\sigma^x|\phi'_{\boldsymbol{k}-}\rangle, \tag{17}$$

and h_1^{ex} is the contribution of the electron and hole excitations,

$$h_1^{\mathrm{ex}} = -U\sum_{\nu}\nu\int \frac{d^2\boldsymbol{k}}{(2\pi)^2}\langle\phi'_{\boldsymbol{k}\nu}|\sigma^x|\phi'_{\boldsymbol{k}\nu}\rangle\delta f_{\boldsymbol{k}\nu}, \tag{18}$$

where $\delta f_{\boldsymbol{k}+} \equiv f_{\boldsymbol{k}+}$ corresponds to electrons and $\delta f_{\boldsymbol{k}-} \equiv 1 - f_{\boldsymbol{k}-}$ to holes.

We begin with h_1^{ex}. Recall that the Floquet states and quasienergies result from the solution of the mean-field Hamiltonian [Supplementary Eq. (13)], and therefore depend (implicitly) on h_1. As we are interested in the regime near the critical value ($U \sim U_{\mathrm{c}}$), where h_1 is small, we expand the states $\{|\phi'_{\boldsymbol{k}\nu}\rangle\}$ in the integrand in Supplementary Eq. (18) in powers of h_1 around $h_1 = 0$. The zeroth-order term in h_1 is proportional to $\langle\phi'_{\boldsymbol{k}\nu}|\sigma^x|\phi'_{\boldsymbol{k}\nu}\rangle\delta f_{\boldsymbol{k}\nu}|_{h_1=0}$. This contribution vanishes, as it consists of the momentum integral over an odd-parity function (arising from the symmetry to reflections of the x-axis, exhibited by $H'_{\mathrm{MF,RWA}}$ at $h_1 = 0$). The contribution to linear order in h_1 includes two terms: the first one is proportional to $h_1\left[\frac{\partial\langle\phi'_{\boldsymbol{k}\nu}|\sigma^x|\phi'_{\boldsymbol{k}\nu}\rangle}{\partial h_1}\delta f_{\boldsymbol{k}\nu}\right]_{h_1=0}$ and the second to $h_1\left[\frac{\partial\delta f_{\boldsymbol{k}\nu}}{\partial h_1}\langle\phi'_{\boldsymbol{k}\nu}|\sigma^x|\phi'_{\boldsymbol{k}\nu}\rangle\right]_{h_1=0}$. In the limit of low filling, the momentum integral over the first term is proportional to the density of particles in the upper band, while the momentum integral over the second term is proportional to the inverse of the density (see below). Therefore, in the low-density limit, considered throughout, we neglect the first term (arising from the dependence of the eigenstates on h_1) with respect to the second one (which captures the change of the distribution function due to h_1).

In order to compute $\frac{\partial\delta f_{\boldsymbol{k}\nu}}{\partial h_1}|_{h_1=0}$, we approximate the distribution function by the Fermi function

$$\delta f_{\boldsymbol{k}\pm} \approx [1 + e^{(\varepsilon_{\boldsymbol{k}} - \Delta_{\mathrm{F}}/2 - \mu_{\mathrm{e/h}})/T_{\mathrm{e/h}}}]^{-1}. \tag{19}$$

Here we used the particle-hole symmetry of the system, where $\varepsilon_{\boldsymbol{k}}$ is the dispersion relation of the upper Floquet band [cf. Supplementary Eq. (7) for $h_1 = 0$ case]. Note that the effective temperature, $T_{\mathrm{e/h}}$, and the effective chemical potential, $\mu_{\mathrm{e/h}}$, must be even functions of h_1, as the setting $h_1 \to -h_1$ inverts the position of the band minimum in the momentum space, but does not change the overall energetics of the system. Therefore, the linear-order dependence of $\delta f_{\boldsymbol{k}\nu}$ on h_1 results predominantly from the dependence of $\varepsilon_{\boldsymbol{k}}$ on h_1. We find this dependence using first-order perturbation theory, $\varepsilon_{\boldsymbol{k}} = \varepsilon_{0,k} + h_1\langle\phi'_{0,\boldsymbol{k}+}|\sigma^x|\phi'_{0,\boldsymbol{k}+}\rangle + \mathcal{O}(h_1^2)$, where $|\phi'_{0,\boldsymbol{k}\nu}\rangle \equiv |\phi'_{\boldsymbol{k}\nu}\rangle|_{h_1=0}$, and $\varepsilon_{0,k} \equiv \varepsilon_{\boldsymbol{k}}|_{h_1=0}$. Finally, using the chain rule, we arrive at $\frac{\partial\delta f_{\boldsymbol{k}\nu}}{\partial h_1}|_{h_1=0} = \frac{\partial\delta f^0_{\boldsymbol{k}\nu}}{\partial\varepsilon_{0,k}}\langle\phi'_{0,\boldsymbol{k}+}|\sigma^x|\phi'_{0,\boldsymbol{k}+}\rangle$,

where $\delta f^0_{k\nu} \equiv \delta f_{\boldsymbol{k}\nu}|_{h_1=0}$ [see Supplementary Eq. (19)]. To make the notations more transparent, throughout we distinguish between rotation-symmetric functions (dependent only on the momentum amplitude) with index k, and functions of momentum amplitude and angle with index $\boldsymbol{k}$.

We substitute the result of the expansion in small h_1 back into Supplementary Eq. (18), yielding

$$h_1^{\rm ex} = -h_1 U \sum_\nu \int \frac{d^2\boldsymbol{k}}{(2\pi)^2} \frac{\partial \delta f^0_{k\nu}}{\partial \varepsilon_{0,k}} |\langle \phi'_{0,\boldsymbol{k}+}|\sigma^x|\phi'_{0,\boldsymbol{k}+}\rangle|^2. \tag{20}$$

To evaluate the integral in Supplementary Eq. (20), we use polar coordinates $\boldsymbol{k} = k(\cos\theta, \sin\theta)$, yielding

$$h_1^{\rm ex} = -h_1 U \sum_\nu \int \frac{kdk}{2\pi} \frac{\partial \delta f^0_{k\nu}}{\partial \varepsilon_{0,k}} \overline{\sigma_k^2}, \tag{21}$$

where $\overline{\sigma_k^2} \equiv \int \frac{d\theta}{2\pi} |\langle \phi'_{0,\boldsymbol{k}+}|\sigma^x|\phi'_{0,\boldsymbol{k}+}\rangle|^2$. To further simplify the expression, we approximate $\overline{\sigma_k^2}$ by its value at the resonance ring, $\overline{\sigma_{k_{\rm R}}^2}$. Corrections to this approximation lead to higher order terms in the density of excitations, and hence are small in the low-density limit. We transform the integral over the magnitude of the momentum k, in Supplementary Eq. (21) to an integral over energy ε, by introducing the density of states $D_{\rm F}(\varepsilon) \equiv \int \frac{d^2\boldsymbol{k}}{(2\pi)^2} \delta(\varepsilon - \varepsilon_{0,k} + \Delta_{\rm F}/2)$. These transformations lead to

$$h_1^{\rm ex} \approx -h_1 U \overline{\sigma_{k_{\rm R}}^2} \sum_{\nu=\pm} \int_0^\infty d\varepsilon D_{\rm F}(\varepsilon) \partial_\varepsilon \delta f^0_{\varepsilon\nu}, \tag{22}$$

where $\delta f^0_{\varepsilon\pm} = [1 + e^{(\varepsilon - \mu_{\rm e/h})/T_{\rm e/h}}]^{-1}$, cf. Supplementary Eq. (19). We estimate the density of states near the band bottom by

$$D_{\rm F}(\varepsilon) \approx D_0 \sqrt{\Delta_{\rm F}/\varepsilon}, \tag{23}$$

where D_0 is a constant depending on the parameters of the Floquet bands. In terms of the bare parameters of the model, we evaluate $D_0 = m_*/2\pi$. We use the eigenstates given in Supplementary Eq. (9) to evaluate, $\overline{\sigma_{k_{\rm R}}^2} = \frac{1}{2}$.

To perform the energy integral in Supplementary Eq. (22), we define a dimensionless integration variable. For $\nu = +$ term, we define $x = \varepsilon/T_{\rm e}$. The integral then reads $\int_0^\infty d\varepsilon D_{\rm F}(\varepsilon)\partial_\varepsilon \delta f^0_{\varepsilon+} = D_{\rm F}(T_{\rm e}) \int_0^\infty \frac{dx}{\sqrt{x}} \partial_x \frac{1}{1+e^{x-(\mu_{\rm e}/T_{\rm e})}} = \sqrt{\pi} D_{\rm F}(T_{\rm e}) {\rm Li}_{-1/2}(-e^{\mu_{\rm e}/T_{\rm e}})$, where ${\rm Li}_s(z)$ is the polylogarithm function[1]. To bring this result into the form of Eq. (10) in the main text, we replace the term proportional to the temperature by $\sqrt{T_{\rm e}} = -n_{\rm e}/[\sqrt{\pi \Delta_{\rm F}} D_0 {\rm Li}_{1/2}(e^{-\mu_{\rm e}/T_{\rm e}})]$. The last relation follows from the definition of the electron density $n_{\rm e} = \int d\varepsilon D_{\rm F}(\varepsilon) \delta f^0_{\varepsilon+} = -\sqrt{\pi} T_{\rm e} D_{\rm F}(T_{\rm e}) {\rm Li}_{1/2}(e^{-\mu_{\rm e}/T_{\rm e}})$. We repeat the same calculation for the lower band ($\nu = -$), to arrive at

$$h_1^{\rm ex} = h_1 U U_{\rm ex}^{-1} \left(\frac{\tilde\Theta(\mu_{\rm e}/T_{\rm e})}{n_{\rm e}/\mathcal{A}_{\rm R}} + \frac{\tilde\Theta(\mu_{\rm h}/T_{\rm h})}{n_{\rm h}/\mathcal{A}_{\rm R}} \right). \tag{24}$$

Here $U_{\rm ex}^{-1} = 2\overline{\sigma_{k_{\rm R}}^2} \Delta_{\rm F} D_0^2/\mathcal{A}_{\rm R}$ and $\tilde\Theta(x) \equiv \frac{\pi}{2} {\rm Li}_{-1/2}(-e^x){\rm Li}_{1/2}(-e^x)$, see Supplementary Fig. 1.

Now, we turn to the evaluation of $h_1^{\rm fb}$ given in Supplementary Eq. (17). In contrast to the integral in Supplementary Eq. (18), which is limited to an area in k-space near the resonance ring, the integral in Supplementary Eq. (17) is defined over the entire Brillouin zone. The dependence on h_1 arises from the dependence of Floquet states $\{|\phi'_{\boldsymbol{k}\nu}\rangle\}$ on h_1. Using first order perturbation theory, we find $|\phi'_{\boldsymbol{k}-}\rangle = |\phi'_{0,\boldsymbol{k}-}\rangle - h_1 \frac{\langle \phi'_{0,\boldsymbol{k}+}|\sigma^x|\phi'_{0,\boldsymbol{k}-}\rangle}{2\varepsilon_k} |\phi'_{0,\boldsymbol{k}+}\rangle$. Employing this expansion of the Floquet states, we express the linear order term in h_1 of Supplementary Eq. (17) as

$$h_1^{\rm fb} = h_1 U \int \frac{d^2\boldsymbol{k}}{(2\pi)^2} \frac{|\langle \phi'_{0,\boldsymbol{k}-}|\sigma^x|\phi'_{0,\boldsymbol{k}+}\rangle|^2}{\varepsilon_k}. \tag{25}$$

This integral is independent of the steady-state distribution [by the definition, see Supplementary Eq. (17)]. We denote the value of this integral by $U_{\rm fb}^{-1} = \int \frac{d^2\boldsymbol{k}}{(2\pi)^2} |\langle \phi'_{0,\boldsymbol{k}-}|\sigma^x|\phi'_{0,\boldsymbol{k}+}\rangle|^2/\varepsilon_k$.

To evaluate $U_{\rm fb}$, we first note that the mean-field Hamiltonian, $H_{\rm MF}(\boldsymbol{k}, t)$ (see Eq. (3) in the main text) describes the system only near the Γ-point, while the integral in the definition of $U_{\rm fb}$ is over the entire Brillouin zone. To address this issue, we impose a cutoff in the integral at the momentum Λ, as a stand-in for the Brillouin zone edge. We evaluate the wavefunctions using Supplementary Eq. (8), where we approximate $\hat{\boldsymbol{z}} \cdot \hat{\boldsymbol{d}}_{\rm F} \approx (k^2/2m_* - \delta E/2)/\varepsilon_k$, with ε_k given by Supplementary Eq. (7). Substituting in Supplementary Eq. (25) we find

$$U_{\rm fb}^{-1} = \frac{m_*}{2\pi} \left[\log\left(\frac{4E_{\rm BW}}{\delta E - k_{\rm R}^2/m_*} \right) - 1 \right], \tag{26}$$

where we denote $E_{\rm BW} = \varepsilon_k|_{k=\Lambda}$.

Summing Supplementary Eqs. (24) and (25) we arrive at

$$h_1 = h_1 U \left[U_{\rm ex}^{-1} \left(\frac{\tilde\Theta(\mu_{\rm e}/T_{\rm e})}{n_{\rm e}/\mathcal{A}_{\rm R}} + \frac{\tilde\Theta(\mu_{\rm h}/T_{\rm h})}{n_{\rm h}/\mathcal{A}_{\rm R}} \right) + U_{\rm fb}^{-1} \right]. \tag{27}$$

Note that h_1 appears on the both sides of Supplementary Eq. (27). Dividing by h_1 and by U, we obtain the expression for $U_{\rm c}$ given in Eq. (10) in the main text.

The calculation outlined in this section helps to find the critical value of U by expanding the self-consistent equation [Eq. (2) in the main text] to the linear order in the order parameter. Expanding this equation to higher orders in h_1 reveals how the order parameter grows as a function of U.

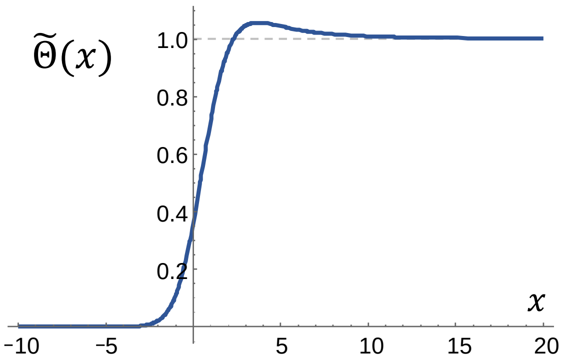

Supplementary Figure 1. **The smeared step function.** A plot of the smeared step function $\widetilde{\Theta}(x) \equiv \frac{\pi}{2}\mathrm{Li}_{-1/2}(-e^{x})\mathrm{Li}_{1/2}(-e^{x})$, appearing in Eq. (10) in the main text.

Supplementary Note 3. THE EXTENDED RATE MODEL

In this section, we give a detailed description of the extended rate model. The results of the model are used to fit the numerical data of $\mu_\mathrm{e}/T_\mathrm{e}$ in Fig. 3 in the main text and to estimate the heating rates in the discussion section. We partially follow the analysis of Ref. 2. Throughout this section, we consider the "paramagnetic phase", with no spontaneous symmetry breaking, corresponding to $U \leq U_\mathrm{c}$. In our analysis, we assume that the system reached a steady state with a low-density of electrons (n_e) in the upper Floquet band (UFB) and holes (n_h) in the lower Floquet band (LFB), $n_\mathrm{e}, n_\mathrm{h} \ll \mathcal{A}_\mathrm{R}$. We verified numerically that the distributions of each of the bands can be well approximated by Fermi functions with effective parameters, see Supplementary Fig. 9.

Here we consider only an electron-doped case ($\Delta n > 0$); the analysis of the hole-doped system is similar due to particle-hole symmetry of the Hamiltonian. We distinguish between two cases: (i) The electrons form a degenerate Fermi gas ($\mu_\mathrm{e}/T_\mathrm{e} \gg 1$) featuring two concentric Fermi surfaces. We refer to this case as the electron Floquet metal (EFM) phase (see Supplementary Fig. 2). In this phase we evaluate energy integrals using the Sommerfeld expansion[3]. (ii) Non-degenerate distribution of electrons ($\mu_\mathrm{e}/T_\mathrm{e} < 0$) referred to as the electron Floquet insulator (EFI) phase. In this phase, we approximate the distribution by the Maxwell's law

$$f_{\varepsilon+} = z_\mathrm{e} e^{-\varepsilon/T_\mathrm{e}}, \tag{28}$$

where $z_\mathrm{e} = e^{-|\mu_\mathrm{e}|/T_\mathrm{e}}$ is the electron fugacity and ε is accounted from the UFB bottom. The two phases are separated by a crossover regime, $\mu_\mathrm{e} \approx T_\mathrm{e}$, where our analysis does not apply. Yet, as we show below, an analytic interpolation between the EFM and the EFI phases gives a good agreement with the numerical data (see Fig. 3 in the main text). We also show below that for the system considered in this paper, the holes always form a non-degenerate distribution in the electron-doped system. Therefore we approximate the distribution of holes by $\bar{f}_{\varepsilon-} \equiv 1 - f_{\varepsilon-}$, where

$$\bar{f}_{\varepsilon-} = z_\mathrm{h} e^{\varepsilon/T_\mathrm{h}}. \tag{29}$$

Here ε is accounted from the LFB top and $z_h = e^{-|\mu_\mathrm{h}|/T_\mathrm{h}}$ is the hole fugacity.

In what follows, we seek four equations for four variables, μ_e, μ_h, T_e, and T_h. Two of the equations [Supplementary Eqs. (34) and (35)] determine the total densities of electrons and holes, n_e and n_h (identical to the derivation of Eq. (7) in the main text). The other two equations [Supplementary Eqs. (44) and (45)], are rate equations for the densities of subpopulations (defined below) of the electron and hole distributions. The rate equations include the key processes leading to the steady-state distribution due to electron-phonon, electron-photon and electron-electron interactions, see Supplementary Fig. 3. Our goal is to find the electron and hole densities and the densities of their subpopulations as functions of the rates in the steady state, and use them to find the chemical potentials and temperatures of the hole and electron distribution. The total densities are related to the parameters of the distribution through

$$n_\mathrm{e} = \int \frac{d^2\boldsymbol{k}}{(2\pi)^2} f_{\boldsymbol{k}+} \quad , \quad n_\mathrm{h} = \int \frac{d^2\boldsymbol{k}}{(2\pi)^2}(1 - f_{\boldsymbol{k}-}). \tag{30}$$

We perform the momentum integral, when the electronic population is deep in the EFM phase, to estimate

$$n_\mathrm{e} \approx 2\mu_\mathrm{e} D_\mathrm{F}(\mu_\mathrm{e}), \tag{31}$$

where D_F is given in Supplementary Eq. (23). In the EFI phase, we perform the integral in Supplementary Eq. (30) using the Maxwell's distribution [see Supplementary Eq. (28)], leading to

$$n_\mathrm{e} \approx z_\mathrm{e}\sqrt{\pi} T_\mathrm{e} D_\mathrm{F}(T_\mathrm{e}). \tag{32}$$

Likewise, we estimate the hole density [using Supplementary Eqs. (30) and (29)] by

$$n_\mathrm{h} \approx z_\mathrm{h}\sqrt{\pi} T_\mathrm{h} D_\mathrm{F}(T_\mathrm{h}). \tag{33}$$

Later in this section, we use the estimates of the densities in terms of the temperature and chemical potential to find the electron and hole temperatures as functions of the parameters of the electronic dispersion in the material, the periodic drive, and the heat-baths.

a. Equations determining the steady-state densities of the electrons and holes

Our first equation relates the total densities of electrons and holes with the doping,

$$n_\mathrm{e} - n_\mathrm{h} = \Delta n. \tag{34}$$

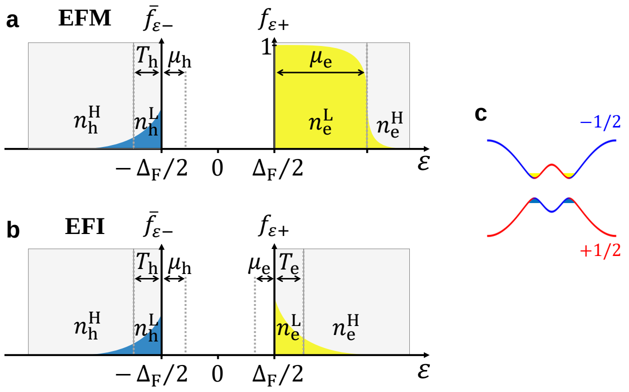

Supplementary Figure 2. **Steady state distributions in the EFM and EFI phases.** The yellow area indicates population of electrons at the bottom of the UFB and the blue area indicates population of holes at the top of the LFB. **a** Distribution in the EFM phase. The electrons exhibit a sharp Fermi-surface at the effective chemical potential μ_e. We denote densities of subpopulations occupying levels below and above the Fermi surface by n_e^L and n_e^H, respectively. **b** Distribution of electrons and holes in the EFI phase. The effective chemical potential of the electrons is in the Floquet gap, giving rise to a Maxwell distribution, given by Supplementary Eq. (28). In this case, the low and high-quasienergy populations occupy states below and above the effective temperature. The population of holes in an electron-doped system is always in the non-degenerate phase described by Supplementary Eq. (29). **c** Schematic drawing of the Floquet bands. The red/blue colored sectors indicates the character of the original valence/conduction bands. The color code helps to visually estimate the power n of the factor $(V/\Omega)^{2n}$ multiplying a scattering rate for the lth order Floquet Umklapp process. The power is given by $n = |l + m_i - m_f|$, where $m_i = 1/2$ when the state before the scattering is in the red sector and $m_i = -1/2$ when it is in the blue sector, similarly, m_f denotes the sector after the scattering.

While the doping sets the difference between the densities of electrons and holes, it does not resolve the density of each of them. Indeed, even at zero doping, non-zero densities of electrons and holes can be created by the drive due to excitation processes. In fact, the populations of the bands are determined by the balance between the processes transferring electrons from the LFB to the UFB (which we will refer to as heating processes), and processes transferring particles from the UFB to LFB (which we will refer to as cooling processes). The heating processes arise from electron-photon, electron-phonon and electron-electron scattering. We capture the effect of each of these processes by a single parameter Γ_ℓ, Γ_s, and Γ_{ee}, cf. Supplementary Fig. 3a-c, which we estimate in Supplementary Note 3 d 1-3.

The interband cooling processes are dominated by electron-hole recombination assisted by phonons. We expect the recombination rate to be proportional to the densities of electrons, n_e and holes, n_h (cf. Supplementary Fig. 3d). Therefore, we estimate $\dot{n}_e|_{cool} = -\Lambda_{inter} n_e n_h$, see Supplementary Note 3 e for the evaluation of Λ_{inter}. The interplay of these processes is captured by the rate equation for n_e,

$$\dot{n}_e = \Gamma_\ell + \Gamma_s + \Gamma_{ee} - \Lambda_{inter} n_e n_h, \tag{35}$$

satisfying $\dot{n}_e = 0$ in the steady state. Note that from the conservation of particles, $\dot{n}_h = -\dot{n}_e$. In the steady state ($\dot{n}_e = 0$), Supplementary Eq. (35) yields

$$n_e n_h = \kappa, \tag{36}$$

where we defined,

$$\kappa \equiv \frac{\Gamma_\ell + \Gamma_s + \Gamma_{ee}}{\Lambda_{inter}}. \tag{37}$$

Combining Supplementary Eqs. (34) and (36), we arrive at the total densities of electrons and holes in the UFB and LFB,

$$n_{e/h} = \sqrt{(\Delta n/2)^2 + \kappa} \pm \Delta n/2, \tag{38}$$

similar to Eq. (7) in the main text.

b. Equations determining the effective temperatures of the electron and hole distributions

Supplementary Eq. (38) yields the total density of electrons and holes, but provides no information on how the particles are distributed within each of the bands. In order to obtain the quasienergy-resolved structure of the distribution in the UFB, we split the electronic population to two subpopulations[2]. We define a subpopulation occupying low-quasienergy levels close to the bottom of the UFB as

$$n_e^L = \int_{\Delta_F/2}^{\Delta_F/2+E_s} D_F(\varepsilon - \Delta_F/2) f_{\varepsilon+} d\varepsilon \tag{39}$$

where D_F is the density of states, given in Supplementary Eq. (23). We furthermore define a complementary subpopulation corresponding to electrons in quasienergy levels not included in Supplementary Eq. (39). The density of this subpopulation is given by $n_e^H = n_e - n_e^L$. In the EFM, we choose $E_s = \mu_e$ and in the EFI $E_s = T_e$, see Supplementary Figs. 2a and b for an illustration. A different choice of E_s in the EFI phase, leads to renormalization of the coefficients in the phenomenological model (see below), but does not change the final result. The splitting into subpopulations makes it possible to study intraband processes in terms of rate equations for the densities of the subpopulations. It is also convenient to define the density of unoccupied states in the low-quasienergy subset of the UFB, denoted by $\bar{n}_e^L$. The definition of $\bar{n}_e^L$ is similar to Supplementary Eq. (39), where we replace $f_{\varepsilon+}$ by $1 - f_{\varepsilon+}$. When the electrons are deep in

the EFM phase, we evaluate their subpopulations [using Supplementary Eq. (39)] by

$$n_{\rm e}^{\rm H} \approx \bar{n}_{\rm e}^{\rm L} \approx \log(2) T_{\rm e} D_{\rm F}(\mu_{\rm e}) \text{ and } n_{\rm e}^{\rm L} \approx n_{\rm e}, \tag{40}$$

where $n_{\rm e}$ is given in Supplementary Eq. (31). In turn, when the electrons exhibit the EFI phase, we evaluate

$$n_{\rm e}^{\rm L} \approx {\rm erf}(1) n_{\rm e},\ n_{\rm e}^{\rm H} \approx {\rm erfc}(1) n_{\rm e} \text{ and } \bar{n}_{\rm e}^{\rm L} \approx 2T_{\rm e} D_{\rm F}(T_{\rm e}), \tag{41}$$

where ${\rm erf}(1) \approx 0.843$, ${\rm erfc}(1) = 1 - {\rm erf}(1)$ and $n_{\rm e}$ is given in Supplementary Eq. (32).

Likewise, we split the distribution of holes in the LFB into two subpopulations. A subpopulation near the top of the LFB within a quasienergy window $\varepsilon \in [-\Delta_{\rm F}/2 - T_{\rm h}, -\Delta_{\rm F}/2]$. The density of this subpopulation is given by

$$n_{\rm h}^{\rm L} = \int_{-\Delta_{\rm F}/2 - T_{\rm h}}^{-\Delta_{\rm F}/2} D_{\rm F}(-\varepsilon - \Delta_{\rm F}/2)(1 - f_{\varepsilon -}) d\varepsilon, \tag{42}$$

and the complementary subpopulation, whose density is $n_{\rm h}^{\rm H} = n_{\rm h} - n_{\rm h}^{\rm L}$. We also define the density of populated states near the top of the LFB, $\bar{n}_{\rm h}^{\rm L}$, defined by Supplementary Eq. (42) with $1 - f_{\varepsilon -}$ replaced by $f_{\varepsilon -}$. Explicitly performing the integral in Supplementary Eq. (42) on the distribution of holes [given in Supplementary Eq. (29)], we evaluate

$$n_{\rm h}^{\rm L} \approx {\rm erf}(1) n_{\rm h},\ n_{\rm h}^{\rm H} \approx {\rm erfc}(1) n_{\rm h} \text{ and } \bar{n}_{\rm h}^{\rm L} \approx 2T_{\rm h} D_{\rm F}(T_{\rm h}), \tag{43}$$

where $n_{\rm h}$ is given in Supplementary Eq. (33).

In what follows, we express $\dot{n}_{\rm e}^{\rm L}$ and $\dot{n}_{\rm h}^{\rm L}$ in terms of the incoming and outgoing rates, and find a balance between them in the steady state by requiring $\dot{n}_{\rm e}^{\rm L} = 0$ and $\dot{n}_{\rm h}^{\rm L} = 0$. We begin with the equation for $\dot{n}_{\rm e}^{\rm L}$. The subpopulations of the UFB are subjected to intraband relaxation processes transferring particles between high- and low-quasienergy sectors within the band. Essentially, these are phonon-assisted electron-hole pair annihilation processes with the rate estimated by $\dot{n}_{\rm e}^{\rm L}|_{\rm relax} = \Lambda_{\rm intra}(T_{\rm e}) n_{\rm e}^{\rm H} \bar{n}_{\rm e}^{\rm L}$ (see Supplementary Fig. 3e). As we show in Supplementary Note 3 f, the relaxation rate in the UFB depends on the temperature of the electrons. The incoming rate of electrons into the low-quasienergy sector is balanced by the phonon-assisted interband recombination rate, serving as a sink of electrons from this sector. The interband recombination rate is proportional to the density of electrons in the low-quasienergy sector in the UFB and density of holes in the LFB, with approximately the same coefficient $\Lambda_{\rm inter}$, as the total interband relaxation appearing in Supplementary Eq. (35). We thus estimate the interband recombination rate by $\dot{n}_{\rm e}^{\rm L}|_{\rm inter} = -\Lambda_{\rm inter} n_{\rm e}^{\rm L} n_{\rm h}$.

Our treatment also includes equilibration between electron and hole distributions by electron-electron scattering. Electron-electron scattering tends to equalize the temperatures of the populations in the UFB and LFB, without changing their total densities (see Supplementary Fig. 3f). As the population of each of the bands is assumed to be in a local equilibrium, which is well-described by the Fermi-function, scattering of two electrons in the same band can not affect it. To account for the interband equilibration, it is convenient to define the average temperature of the UFB and LFB populations $T = (T_{\rm e} + T_{\rm h})/2$ and their temperature difference, $\Delta T = T_{\rm e} - T_{\rm h}$.

First, assume that the electrons and holes have the same temperature ($\Delta T = 0$). In this case, the thermalization rate is zero, as the populations are already in equilibrium. Next, consider a small temperature difference of the populations, ΔT. If $\Delta T > 0$, the electron-electron scattering induces electron-hole pair annihilation in the UFB and electron-hole pair creation in the LFB, effectively cooling down the UFB and heating up the LFB. In contrast, when $\Delta T < 0$, electron-hole pairs are created in the UFB and annihilated in the LFB. When the thermalization is significant, we expect the temperatures of the two bands to be almost the same, i.e., $|\Delta T| \ll T$. Such pair creation and annihilation in the two bands occurs in a quasienergy window of width $\sim T$. Notice that in the numerical simulation (Figs. 2 and 3 in the main text), the equilibration between electron and hole distributions due to electron-electron scattering was not taken in account. We later justify this (see Supplementary Note 3 h 2) by showing that this effect does not significantly affect the majority population.

Following from the above arguments, we expect the electron-electron scattering rate to be linear in ΔT to the leading order. We estimate the equilibration rate by $\dot{n}_{\rm e}^{\rm L}|_{\rm ee} = \gamma_{\rm ee} \Delta T n_{\rm e}^{\rm H} \bar{n}_{\rm e}^{\rm L} n_{\rm h}^{\rm H} \bar{n}_{\rm h}^{\rm L}$. This rate serves as a source of particles for the low-quasienergy sector in the UFB when $\Delta T > 0$, and a sink when $\Delta T < 0$. It is proportional to the densities in the UFB of occupied states (above $E_{\rm s}$) and unoccupied states (below $E_{\rm s}$) available for annihilation, and densities in the LFB of occupied states (above $-\Delta_{\rm F}/2 - T_{\rm h}$) and unoccupied states (below $-\Delta_{\rm F}/2 - T_{\rm h}$) available for a pair creation. A further justification for this form will be discussed in Supplementary Note 3 g, where we estimate the value of $\gamma_{\rm ee}$ in terms of the system's parameters. Combining all the terms above, we arrive at the full rate equation for $n_{\rm e}^{\rm L}$, which reads

$$\dot{n}_{\rm e}^{\rm L} = \Lambda_{\rm intra}(T_{\rm e}) n_{\rm e}^{\rm H} \bar{n}_{\rm e}^{\rm L} - \Lambda_{\rm inter} n_{\rm e}^{\rm L} n_{\rm h} + \gamma_{\rm ee} \Delta T n_{\rm e}^{\rm H} \bar{n}_{\rm e}^{\rm L} n_{\rm h}^{\rm H} \bar{n}_{\rm h}^{\rm L}. \tag{44}$$

Our fourth equation describes thermalization and relaxation in the LFB, expressed through an equation for $\dot{n}_{\rm h}^{\rm L}$. The intraband and interband rates are estimated by $\dot{n}_{\rm h}^{\rm L}|_{\rm intra} = \Lambda_{\rm intra}(T_{\rm h}) n_{\rm h}^{\rm H} \bar{n}_{\rm h}^{\rm L}$ and $\dot{n}_{\rm h}^{\rm L}|_{\rm inter} = -\Lambda_{\rm inter} n_{\rm h}^{\rm L} n_{\rm e}$. Here we approximate $\Lambda_{\rm inter}$ and $\Lambda_{\rm intra}$ by the same coefficients as in Supplementary Eq. (44) evaluated at the hole temperature $T_{\rm h}$, as follows from the particle-hole symmetry of the Hamiltonian. In analogy to the discussion above Supplementary Eq. (44), we estimate the electron-electron thermalization rate by $\dot{n}_{\rm h}^{\rm L}|_{\rm ee} = -\gamma_{\rm ee} \Delta T n_{\rm h}^{\rm H} \bar{n}_{\rm h}^{\rm L} n_{\rm e}^{\rm H} \bar{n}_{\rm e}^{\rm L}$. Combining all the incoming and outgoing rates we arrive at the full rate equation for

$n_{\rm h}^{\rm L}$, which reads

$$\dot{n}_{\rm h}^{\rm L} = \Lambda_{\rm intra}(T_{\rm h}) n_{\rm h}^{\rm H} \bar{n}_{\rm h}^{\rm L} - \Lambda_{\rm inter} n_{\rm h}^{\rm L} n_{\rm e} - \gamma_{\rm ee} \Delta T n_{\rm h}^{\rm H} \bar{n}_{\rm h}^{\rm L} n_{\rm e}^{\rm H} \bar{n}_{\rm e}^{\rm L}. \tag{45}$$

In order to solve Supplementary Eqs. (44) and (45) for the chemical potentials and effective temperatures in the steady state [for total densities $n_{\rm e}$ and $n_{\rm h}$ fixed by Supplementary Eq. (38)], we first need to estimate how the densities and the coefficients appearing in these equations (such as $\Lambda_{\rm inter}$, $\Lambda_{\rm intra}$ and $\gamma_{\rm ee}$) depend on the parameters of the steady state. In the sections below, we perform these estimations. In Supplementary Note 3 h, we extract the expressions for the chemical potentials and temperatures as a function of κ, Δn and the parameters of the system.

c. Evaluation of the rates and Floquet-Fermi's golden rule

Before we evaluate the scattering rates for the processes presented in Supplementary Fig. 3, we first review the Fermi's golden rule for transitions between Floquet states[4,5]. For brevity, we use a notation $\boldsymbol{k}\nu$ to indicate a state with momentum $\boldsymbol{k}$ and Floquet band ν.

We first discuss the rate for scattering of an electron between states $\boldsymbol{k}\nu$ and $\boldsymbol{k}'\nu'$ due to a collision with a phonon ($p = {\rm s}$) or photon ($p = \ell$) [resulting from the coupling Hamiltonian in Eq. (5) in the main text]. We assume the phonon bath is at zero temperature. The scattering rate is given by

$$\begin{aligned}(\dot{f}_{\boldsymbol{k}\nu})_{p,\boldsymbol{k}'\nu'} = 2\pi \sum_l \mathcal{P}_p^{(l)}(\boldsymbol{k}\nu, \boldsymbol{k}'\nu') \times \\ [\rho_p(\boldsymbol{q}, \omega_l) F(\boldsymbol{k}'\nu', \boldsymbol{k}\nu) - \rho_p(\boldsymbol{q}, -\omega_l) F(\boldsymbol{k}\nu, \boldsymbol{k}'\nu')],\end{aligned} \tag{46}$$

where $\mathcal{P}_p^{(l)}(\boldsymbol{k}\nu, \boldsymbol{k}'\nu') = \left|\sum_m \langle \phi_{\boldsymbol{k}'\nu'}^{m+l} | \mathcal{M}_p(\boldsymbol{q}, \omega_l) | \phi_{\boldsymbol{k}\nu}^m \rangle\right|^2$ and

$$F(\boldsymbol{k}\nu, \boldsymbol{k}'\nu') = f_{\boldsymbol{k}\nu} \bar{f}_{\boldsymbol{k}'\nu'}, \tag{47}$$

where $\bar{f} \equiv 1 - f$. The in-plane momentum and energy transfers are given by $\boldsymbol{q} = \boldsymbol{k}' - \boldsymbol{k}$ and $\omega_l = \varepsilon_{\boldsymbol{k}'\nu'} - \varepsilon_{\boldsymbol{k}\nu} + l\Omega$. We consider the following electron-phonon coupling

$$\mathcal{M}_{\rm s}(\boldsymbol{q}, \omega) = g_{\rm s} |\boldsymbol{q}| / \sqrt{\omega} \mathbb{1}, \tag{48}$$

where $\mathbb{1}$ is the identity matrix, and consider electron-photon coupling of two polarizations,

$$\mathcal{M}_\ell^{(1)} = g_\ell \sigma^x \quad , \quad \mathcal{M}_\ell^{(2)} = g_\ell \sigma^y. \tag{49}$$

We take the density of states of three-dimensional phonons as a function of the in-plane momentum $\boldsymbol{q}$ (the in-plane momentum of the phonon is the momentum transfer of the electron), $\rho_{\rm s}(\omega, \boldsymbol{q}) = \rho_{\rm s}^0 \omega / \sqrt{\omega^2 - v_{\rm s}^2 |\boldsymbol{q}|^2}$ when $\omega > v_{\rm s}|\boldsymbol{q}|$ and zero otherwise. For the heat bath of photons, we consider $\rho_\ell(\omega, \boldsymbol{q}) = \delta^{(2)}(\boldsymbol{q}) \rho_\ell^0$.

The scattering rate due to electron-electron interaction depends on occupations of four electronic states. Consider an event in which two electrons occupying states $\boldsymbol{k}\nu$ and $\boldsymbol{p}\mu$, scatter into $\boldsymbol{k}'\nu'$ and $\boldsymbol{p}'\mu'$. The net rate of such an event and its reversed process reads

$$\begin{aligned}(\dot{f}_{\boldsymbol{k}\nu})_{\rm ee} = 2\pi \sum_l \mathcal{P}_{\rm ee}^{(l)}(\boldsymbol{k}\nu, \boldsymbol{p}\mu; \boldsymbol{k}'\nu', \boldsymbol{p}'\mu') \times \\ F_{\rm ee}(\boldsymbol{k}\nu, \boldsymbol{p}\mu; \boldsymbol{k}'\nu', \boldsymbol{p}'\mu') \delta(\Delta\varepsilon_1 + \Delta\varepsilon_2 + l\Omega),\end{aligned} \tag{50}$$

where $\mathcal{P}_{\rm ee}^{(l)} = |\sum_{jmn} \langle \phi_{\boldsymbol{k}\nu}^{l-j+m} \phi_{\boldsymbol{p}\mu}^{j+n} | \hat{\mathcal{H}}_{\rm int} | \phi_{\boldsymbol{k}'\nu'}^m \phi_{\boldsymbol{p}'\mu'}^n \rangle|^2$,

$$F_{\rm ee} = f_{\boldsymbol{k}'\nu'} \bar{f}_{\boldsymbol{k}\nu} f_{\boldsymbol{p}'\mu'} \bar{f}_{\boldsymbol{p}\mu} - f_{\boldsymbol{k}\nu} \bar{f}_{\boldsymbol{k}'\nu'} f_{\boldsymbol{p}\mu} \bar{f}_{\boldsymbol{p}'\mu'}, \tag{51}$$

and $\Delta\varepsilon_1 = \varepsilon_{\boldsymbol{k}'\nu'} - \varepsilon_{\boldsymbol{k}\nu}$, $\Delta\varepsilon_2 = \varepsilon_{\boldsymbol{p}'\mu'} - \varepsilon_{\boldsymbol{p}\mu}$. Throughout, we consider contact interactions, $\hat{\mathcal{H}}_{\rm int} = \sum_{\boldsymbol{k}_1\boldsymbol{k}_2\boldsymbol{k}_3} (U/a^2) \hat{c}_{\boldsymbol{k}_1+\boldsymbol{k}_3\uparrow}^\dagger \hat{c}_{\boldsymbol{k}_1\uparrow} \hat{c}_{\boldsymbol{k}_2-\boldsymbol{k}_3\downarrow}^\dagger \hat{c}_{\boldsymbol{k}_2\downarrow}$, where $\hat{c}_{\boldsymbol{k}\uparrow(\downarrow)}^\dagger$ creates an electron of momentum $\boldsymbol{k}$ and pseudospin $\uparrow$ ($\downarrow$) [see discussion below Eq. (1) in the main text]. Using the above form of $\hat{\mathcal{H}}_{\rm int}$, the expression for the squared matrix element can be written as

$$\begin{aligned}\mathcal{P}_{\rm ee}^{(l)} = \frac{U^2}{4a^4} | \sum_{jmn} \langle \phi_{\boldsymbol{k}\nu}^{l-j+m} | \phi_{\boldsymbol{k}'\nu'}^m \rangle \langle \phi_{\boldsymbol{p}\mu}^{j+n} | \phi_{\boldsymbol{p}'\mu'}^n \rangle - \\ - \sum_{\alpha=x,y,z} \langle \phi_{\boldsymbol{k}\nu}^{l-j+m} | \sigma^\alpha | \phi_{\boldsymbol{k}'\nu'}^m \rangle \langle \phi_{\boldsymbol{p}\mu}^{j+n} | \sigma^\alpha | \phi_{\boldsymbol{p}'\mu'}^n \rangle |^2,\end{aligned} \tag{52}$$

when $\boldsymbol{k} + \boldsymbol{p} = \boldsymbol{k}' + \boldsymbol{p}'$ and $\mathcal{P}_{\rm ee}^{(l)} = 0$ otherwise. Here σ^α is a Pauli matrix in the pseudospin basis. Clearly, the RHS of Supplementary Eq. (52) vanishes when the two colliding electrons have parallel pseudospins, and is maximal when the pseudospins are antiparallel. This property directly follows from the contact interactions between the electrons and the Pauli principle. Once we established the Floquet-Fermi's golden rule formalism, we are in the position to evaluate the heating, cooling and thermalization rates.

d. Heating rates

We begin our discussion with heating processes, transferring electrons from the LFB to UFB. For zero-temperature heat baths, energy required for the excitations is provided by the Floquet-Umklapp mechanism, dominated by $l = 1$ terms in Supplementary Eqs. (46) and (50). In the estimation of heating rates we will approximate $f_{\boldsymbol{k}-} \approx 1$ and $f_{\boldsymbol{k}+} \approx 0$, which is exact in the limit $\kappa \to 0$. As the majority of excitation processes occur away from the resonance ring, we simplify the analysis by taking the limit $V/\Omega \to 0$. In this limit, the Floquet wavefunctions are not analytic at $|\boldsymbol{k}| = k_{\rm R}$, yet obtain a simple structure away from the resonance ring. The zeroth order in (V/Ω) reads

$$|\phi_{\boldsymbol{k}\pm}(t)\rangle = e^{\frac{i\Omega t}{2}} \begin{cases} |\Psi_{\boldsymbol{k}\mp}\rangle e^{\pm\frac{i\Omega t}{2}} & , |\boldsymbol{k}| < k_{\rm R} \\ |\Psi_{\boldsymbol{k}\pm}\rangle e^{\mp\frac{i\Omega t}{2}} & , |\boldsymbol{k}| > k_{\rm R} \end{cases}, \tag{53}$$

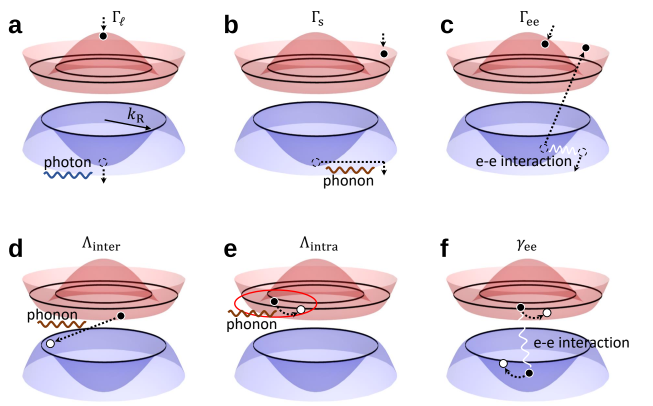

Supplementary Figure 3. **Key processes contributing to the steady-state distribution.** We consider an EFM phase for the electrons in the UFB, associated with two concentric Fermi surfaces (represented by a double black ring), and a non-degenerate distribution of holes in the LFB, with a maximum of the distribution shown by a single black ring. We assume $U \lesssim U_c$, such that the rotational symmetry is unbroken. The heating processes are summarized in panels **a**, **b**, and **c**; Panels **d**, **e**, and **f** demonstrate cooling and thermalization processes. **a** Photon-mediated excitation processes, dominant inside the resonance ring, with approximately zero-momentum transfer. **b** Phonon-mediated excitations, predominantly transferring electrons between states inside and outside the resonance ring. **c** Floquet-Auger heating, corresponding to a transition of two electrons occupying the interior and exterior of the resonance ring, to states outside and inside the resonance ring, respectively (see Supplementary Fig. 4). **d** Phonon-assisted interband recombination of electrons occupying the Fermi sea in the UFB and holes in the LFB. Assuming a large Floquet gap ($\Delta_F \gg 2k_R v_s$) the phonon density of states supports scattering between states in the UFB and LFB of any two points close to the resonance ring. **e** Phonon-assisted intraband relaxation. In the low-temperature limit, $T < 2k_R v_s$, the phonon density of states only allows for small-momentum transitions with a momentum transfer below $\sim T/v_s$. Therefore, the allowed states to scatter into are within a circle of radius $\sim T/v_s$ centered around the initial electron position (indicated by the red circle). **f** Intraband thermalization by electron-electron interactions. The main role of these processes is equilibration of temperatures of the electron and hole distributions. The total density of particles in each of the bands after the collision is preserved.

where $|\Psi_{\boldsymbol{k}\pm}\rangle$ are the eigenstates of $H_0(\boldsymbol{k})$ [see Supplementary Eq. (1)], given by

$$|\Psi_{\boldsymbol{k}\pm}\rangle = \frac{1}{\sqrt{2}}\left(\sqrt{1 \pm \hat{\boldsymbol{z}}\cdot\hat{\boldsymbol{d}}(\boldsymbol{k})}|\uparrow\rangle \mp e^{i\theta_{\boldsymbol{k}}}\sqrt{1 \mp \hat{\boldsymbol{z}}\cdot\hat{\boldsymbol{d}}(\boldsymbol{k})}|\downarrow\rangle\right). \tag{54}$$

To determine the power of V/Ω multiplying the scattering rates for the higher order terms, we use a method described in Supplementary Fig. 2c. Note, that these approximate expressions of the wavefunctions can not be used to analyze the distributions near the Floquet gap. Therefore, we use a different approximation when we discuss relaxation and thermalization processes in Supplementary Note 3 e-g.

1. *Photon-assisted excitation rate*

We begin with the photon-assisted interband excitations. These are vertical Floquet-Umklapp processes [the initial and final momentum of the photons are identical, see the definition of the photon density of states below Supplementary Eq. (49)], predominantly transferring electrons inside the resonance ring. Processes outside the ring can be neglected in the limit $V/\Omega \to 0$, as they are suppressed by a factor of $\sim (V/\Omega)^4$ (see Supplementary Fig. 2c for an explanation). In what follows, we estimate the dominant contribution, illustrated in Supplementary Fig. 3a.

We define the rate of change (ROC) of the density in the UFB as $\dot{n}_e|_{\text{photon}} = \Gamma_\ell$, where

$$\Gamma_\ell = \int \frac{a^2 d^2\boldsymbol{k} d^2\boldsymbol{k}'}{(2\pi)^4}(\dot{f}_{\boldsymbol{k}+})_{\ell,\boldsymbol{k}'-}. \tag{55}$$

We approximate $(\dot{f}_{\boldsymbol{k}+})_{\ell,\boldsymbol{k}'-}$ [given in Supplementary Eq. (46)] by $(\dot{f}_{\boldsymbol{k}+})_{\ell,\boldsymbol{k}'-} \approx 2\pi\mathcal{P}^{(1)}_\ell(\boldsymbol{k}+,\boldsymbol{k}-)\rho^0_\ell\delta(\boldsymbol{k}-\boldsymbol{k}')$, when $|\boldsymbol{k}| < k_R$ and zero otherwise. The sum of squared matrix elements for two photon polarizations [Supplementary Eq. (49)] evaluated in the states $|\Psi_{\boldsymbol{k}\nu}\rangle$ [Supplementary Eq. (53)] inside the resonance ring ($|\boldsymbol{k}| < k_R$) is given by $\mathcal{P}^{(1)}_\ell(\boldsymbol{k}+,\boldsymbol{k}-) = \sum_{i=1,2}|\langle\Psi_{\boldsymbol{k}-}|\mathcal{M}^{(i)}_\ell|\Psi_{\boldsymbol{k}+}\rangle|^2 = g^2_\ell(1+\hat{\boldsymbol{z}}\cdot\hat{\boldsymbol{d}}(\boldsymbol{k}))$. We further approximate $\hat{\boldsymbol{z}}\cdot\hat{\boldsymbol{d}} \approx 1$ to leading order in $\lambda_0 k_R/E_g$, yielding $\mathcal{P}^{(1)}_\ell \approx 2g^2_\ell$ and perform the trivial momentum integral in Supplementary Eq. (55) to arrive at

$$\Gamma_\ell = g^2_\ell\rho^0_\ell\mathcal{A}_R a^2/(4\pi^3). \tag{56}$$

Recall that $\mathcal{A}_R = \pi k^2_R$ is the area in the reciprocal space enclosed by the resonance ring.

2. *Phonon-assisted excitation rate*

Next, we estimate the phonon-assisted excitation rate (for an illustration see Supplementary Fig. 3b). The ROC of the density in the UFB due to phonon-assisted excitation, $\dot{n}_e|_{\text{phonon}} = \Gamma_s$, reads

$$\Gamma_s = \int \frac{a^2 d^2\boldsymbol{k} d^2\boldsymbol{k}'}{(2\pi)^4}(\dot{f}_{\boldsymbol{k}+})_{s,\boldsymbol{k}'-}, \tag{57}$$

where we use the approximate expression

$$(\dot{f}_{\boldsymbol{k}+})_{s,\boldsymbol{k}'-} \approx 2\pi\mathcal{P}^{(1)}_s(\boldsymbol{k}+,\boldsymbol{k}'-)\rho_s(\boldsymbol{k}-\boldsymbol{k}',\omega), \tag{58}$$

and $\omega = \varepsilon_{\boldsymbol{k}'-} - \varepsilon_{\boldsymbol{k}+} + \Omega$, see Supplementary Eq. (46). We evaluate the expectation value in $\mathcal{P}^{(1)}_s$ [defined below

Supplementary Eq. (46)] using the electron-phonon coupling [Supplementary Eq. (48)] and the Floquet states given in Supplementary Eq. (53). The result splits to four different rates, depending whether the momenta $\boldsymbol{k}$ and $\boldsymbol{k}'$ are inside or outside the resonance ring.

First, consider transitions where both momenta $\boldsymbol{k}$ and $\boldsymbol{k}'$ are inside the resonance ring. The rate of such transitions is suppressed by $\sim (\lambda_0 k_{\mathrm{R}}/E_{\mathrm{g}})^2$ as the electron-phonon coupling [which we assume has a diagonal form in the pseudospin basis, see Supplementary Eq. (48)] connects almost orthogonal pseudospin states. Furthermore, transitions inside the resonance ring are unfavorable as the electron-phonon coupling favours short-wavelength phonons, while the wavelength corresponding to the momentum transfer inside the resonance ring is at least $\sim (2k_{\mathrm{R}})^{-1}$. Therefore, in what follows we neglect transitions where both momenta are inside the resonance ring.

Next, we consider transitions for which $\boldsymbol{k}'$ is inside and $\boldsymbol{k}$ is outside the resonance ring (or otherwise, $\boldsymbol{k}'$ outside and $\boldsymbol{k}$ inside the resonance ring). These processes require an absorption of a drive-photon leading to suppression of their rate by a factor of $(V/\Omega)^2$ [see Supplementary Fig. 2c for explanation]. Assuming a narrow-band semiconductor (analogous to the assumption in our numerical analysis, see Tab. 1) we approximate the energy transfer, appearing in the definition of $\mathcal{P}_{\mathrm{s}}^{(1)}$ and ρ_{s} in Supplementary Eq. (58), by $\omega \sim \Omega$, leading to $\mathcal{P}_{\mathrm{s}}^{(1)}(\boldsymbol{k}+, \boldsymbol{k}'-) \approx \frac{g_{\mathrm{s}}^2 |\boldsymbol{k}-\boldsymbol{k}'|^2}{\Omega} \left(\frac{V}{\Omega}\right)^2$ [cf. Supplementary Eq. (48)] and $\rho_{\mathrm{s}}(\boldsymbol{q}, \Omega) \approx \rho_{\mathrm{s}}^0$. Explicitly integrating over $(\dot{f}_{\boldsymbol{k}+})_{\mathrm{s},\boldsymbol{k}'-}$ in Supplementary Eq. (57), we arrive at

$$\Gamma_{\mathrm{s}} \approx \frac{g_{\mathrm{s}}^2 \rho_{\mathrm{s}}^0 a^2 \mathcal{A}_{\mathrm{R}} \Lambda^4}{8\pi^2 \Omega} \left(\frac{V}{\Omega}\right)^2, \tag{59}$$

where $\Lambda \sim \pi/a$ is the large momentum cutoff for phonon transitions. Note that the processes considered here, involve collisions with large-energy phonons (of energy $\sim \Omega$). Such phonons might not be supported in materials in which the Debye frequency is smaller than Ω.

An alternative scenario, which describes many periodically driven semiconductor systems, is the case of a large electronic bandwidth compared to the drive frequency. In this case, the bandstructure supports transitions transferring electrons between momentum states inside and outside the resonance ring (similar to the scenario discussed above), assisted by low-energy phonons. In particular, the allowed energy of the phonon is within the range $\omega \in [\omega_0, \omega_{\mathrm{D}}]$, where ω_{D} is the Debye energy of the semiconductor and ω_0 is the minimal energy allowed by the kinematic constraints. For simplicity, we assume the parabolic dispersion near the Γ-point (with an effective mass m_*) extends to energies $\gtrsim \Omega$. We also assume the limit $k_{\mathrm{R}}/\sqrt{2m_*\Omega} \to 0$. In this limit, the momentum transfer $|\boldsymbol{q}| = |\boldsymbol{k} - \boldsymbol{k}'|$, approximately equals the momentum of the electron after the transition, $|\boldsymbol{k}|$. It follows from the above, the minimal allowed phonon energy ω_0 is given by $\omega_0 = v_{\mathrm{s}}|\boldsymbol{q}_0|$, where $|\boldsymbol{q}_0| = \sqrt{2m_*(\Omega - \omega_0)}$ is the maximal allowed momentum transfer in the process.

To evaluate $\mathcal{P}_{\mathrm{s}}^{(1)}(\boldsymbol{k}+, \boldsymbol{k}'-)$ and the density of states, ρ_{s} [appearing in Supplementary Eq. (58)], we estimate the energy transfer by $\omega \approx \Omega - \varepsilon_{\boldsymbol{k}+}$. Recall that $\boldsymbol{k}$ denotes momentum far from the resonance ring, where the dispersion is approximately parabolic, $\varepsilon_{\boldsymbol{k}+} \approx |\boldsymbol{k}|^2/(2m_*)$. Therefore, we approximate $\mathcal{P}_{\mathrm{s}}^{(1)}(\boldsymbol{k}+, \boldsymbol{k}'-) \approx \frac{g_{\mathrm{s}}^2 |\boldsymbol{k}|^2}{\Omega - \varepsilon_{\boldsymbol{k}+}} \left(\frac{V}{\Omega}\right)^2$ and $\rho_{\mathrm{s}} \approx \frac{\rho_{\mathrm{s}}^0(\Omega - \varepsilon_{\boldsymbol{k}+})}{\sqrt{(\Omega - \varepsilon_{\boldsymbol{k}+})^2 - v_{\mathrm{s}}^2 |\boldsymbol{k}|^2}}$.

We are now ready to evaluate the integral in Supplementary Eq. (57). The $\boldsymbol{k}'$-integral is in the domain $|\boldsymbol{k}'| < k_{\mathrm{R}}$. The integral over $\boldsymbol{k}$ is performed in the range $|\boldsymbol{k}| \in [|\boldsymbol{q}_D|, |\boldsymbol{q}_0|]$, where $\boldsymbol{q}_D$ is found from $\omega_D = \Omega - \varepsilon_{\boldsymbol{q}_D+}$ (leading to $|\boldsymbol{q}_D| = \sqrt{2m_*(\Omega - \omega_{\mathrm{D}})}$.) Transforming to polar coordinates where $k = |\boldsymbol{k}|$ and $k' = |\boldsymbol{k}'|$, we obtain $\Gamma_{\mathrm{s}} = \frac{g_{\mathrm{s}}^2 \rho_{\mathrm{s}}^0 a^2}{\pi} \left(\frac{V}{\Omega}\right)^2 \int_0^{k_{\mathrm{R}}} dk' k' \int_{|\boldsymbol{q}_D|}^{|\boldsymbol{q}_0|} dk k^3 / \sqrt{(\Omega - \varepsilon_{\boldsymbol{k}+})^2 - v_{\mathrm{s}}^2 k^2}$. Explicitly integrating over k and k' in the limit $\Omega \gg \omega_{\mathrm{D}} \gg m_* v_{\mathrm{s}}^2$, we arrive at

$$\Gamma_{\mathrm{s}} \approx \frac{2c_{\mathrm{D}} g_{\mathrm{s}}^2 \rho_{\mathrm{s}}^0 a^2 \mathcal{A}_{\mathrm{R}} m_*^2 \Omega}{\pi^2} \left(\frac{V}{\Omega}\right)^2, \tag{60}$$

where $c_{\mathrm{D}} = \log\left(\frac{\sqrt{2m_* v_{\mathrm{s}}^2 \Omega}}{\omega_{\mathrm{D}} - \sqrt{\omega_{\mathrm{D}}^2 - 2m_* v_{\mathrm{s}}^2 \Omega}}\right)$.

Finally, consider processes where both $\boldsymbol{k}$ and $\boldsymbol{k}'$ are outside the resonance ring. These processes are suppressed by $(V/\Omega)^4$ as they require absorption of two virtual photons [see Supplementary Fig. 2c]. Furthermore, they will be suppressed by $\sim (2m_*\lambda_0/\Lambda)^2$ as such transitions connect almost orthogonal pseudospin states. We thus neglect the contribution of these processes to the phonon-assisted excitation rate.

3. *Floquet-Auger excitation rate*

Here, we estimate the rate of transfer of electrons from the LFB to the UFB due to electron-electron scattering. The main processes contributing to such transfer are electron-pair excitations from the LFB to UFB[6]. For the estimation of this rate, we assume a state with a full LFB and empty UFB (we therefore neglect processes which result in net transfer of electrons from the UFB to the LFB). The leading processes resulting in a transfer of two electrons from the LFB to the UFB involve a change of the total quasienergy by Ω via the Floquet-Umklapp mechanism (illustrated in Supplementary Fig. 3c). The ROC of the density due to these processes is given by $\dot{n}_{\mathrm{e}}|_{\mathrm{ee}} = \Gamma_{\mathrm{ee}}$, where

$$\Gamma_{\mathrm{ee}} = 2 \int \frac{a^4 d^2\boldsymbol{k} d^2\boldsymbol{p} d^2\boldsymbol{q}}{(2\pi)^6} (\dot{f}_{\boldsymbol{k}+})_{\mathrm{ee}}. \tag{61}$$

Here $\boldsymbol{k}$ and $\boldsymbol{p}$ are the momenta of the final states in the UFB and $\boldsymbol{k}' = \boldsymbol{k} + \boldsymbol{q}$, $\boldsymbol{p}' = \boldsymbol{p} - \boldsymbol{q}$ denote the initial momenta in the LFB; the factor 2 accounts for a pair of electrons excited in each collision. We estimate $(\dot{f}_{\boldsymbol{k}+})_{\mathrm{ee}}$, given in Supplementary Eq. (50), by

a

Process	Contribution to Γ_{ee}
$ii \to ii$	Kinematically blocked
$ii \to io$	Kinematically blocked
$ii \to oo$	$\sim (\lambda_0 k_R/E_g)^2 (V/\Omega)^2$
$io \to ii$	Kinematically blocked
$io \to io$	$\sim (V/\Omega)^2$
$io \to oo$	$\sim (V/\Omega)^4$
$oo \to ii$	$\sim (\lambda_0 k_R/E_g)^2 (V/\Omega)^2$
$oo \to io$	$\sim (V/\Omega)^4$
$oo \to oo$	$\sim (V/\Omega)^6$

b

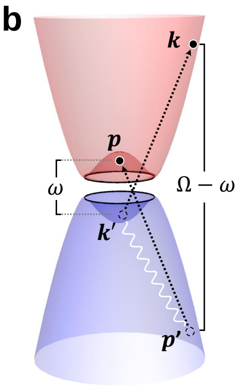

Supplementary Figure 4. **Scattering processes contributing to the Floquet-Auger excitation rate. a** Summary of possible scattering processes and their estimated contribution to $\Gamma_{\rm ee}$, divided to cases. The first column shows the possible types of processes; i and o indicate momenta inside and outside the resonance ring. The first two letters correspond to initial momenta of the two electrons (in the LFB) and the last two letters correspond to momenta after the collision (in the UFB.) The second column shows the estimated weight of each process in terms of powers of (V/Ω). We denote by "kinematically-blocked", processes that can not satisfy the energy and momentum conservation conditions due to the bandstructure constraints. **b** Schematic illustration of the dominant collision, $io \to io$, highlighted in panel **a**. The momenta $\boldsymbol{k}'$ and $\boldsymbol{p}'$ denote the states of two electrons in the LFB before the collision, and the momenta $\boldsymbol{k}$ and $\boldsymbol{p}$ denotes the states in the UFB after the collision. The energy differences, ω and $\Omega - \omega$ are defined as $\omega = \varepsilon_{\boldsymbol{p}+} - \varepsilon_{\boldsymbol{k}'-}$ and $\Omega - \omega = \varepsilon_{\boldsymbol{k}+} - \varepsilon_{\boldsymbol{p}'-}$.

$(\dot{f}_{\boldsymbol{k}+})_{\rm ee} \approx 2\pi \mathcal{P}^{(1)}_{\rm ee}(\boldsymbol{k}+, \boldsymbol{p}+, \boldsymbol{k}'-, \boldsymbol{p}'-)\delta(\Delta\varepsilon_1 + \Delta\varepsilon_2 + \Omega)$ [see definitions of $\Delta\varepsilon_{1,2}$ below Supplementary Eq. (50)], and evaluate $\mathcal{P}^{(1)}_{\rm ee}$ using Supplementary Eq. (52) and the approximate Floquet wavefunctions given in Supplementary Eq. (53). As the approximate wavefunctions are non-analytic at $|\boldsymbol{k}| = k_{\rm R}$, we divide the analysis of the scattering rate to 9 distinct cases. In each case, the momenta $\boldsymbol{k}$, $\boldsymbol{k}'$, $\boldsymbol{p}$ and $\boldsymbol{p}'$ are either inside or outside the resonance ring.

Supplementary Fig. 4a shows a table of all possible cases and their estimated contribution to $\Gamma_{\rm ee}$. We consider the limit $\delta E \ll \Omega$ which kinematically constraints some of the processes. For example, processes where the two initial and the two final momenta are inside the resonance ring (corresponding to $ii \to ii$ in Supplementary Fig. 4a) are kinematically blocked, as there is no such process for which the total energy change (which is limited by $2\delta E$ by the Floquet bandstructure) can be equal Ω. Processes corresponding to $ii \to oo$ and $oo \to ii$ involve scattering of two electrons starting and ending in the same band in terms of the undriven band structure, cf. Supplementary Eq. (53). These processes are Pauli-suppressed by a factor of $\sim (\lambda_0 k_{\rm R}/E_{\rm g})^2$ as the pseudospins of the electrons involved in the collision are almost parallel, see discussion following Supplementary Eq. (52).

The most significant contribution to the rate [proportional to $(V/\Omega)^2$] corresponds to transitions of two electrons initially occupying one state inside the resonance ring and one state outside of it, scattered into two states in the UFB where one is inside and one is outside the resonance ring (denoted by $io \to io$ in Supplementary Fig. 4a). An illustration of such a process is shown in Supplementary Fig. 4b. We choose the momenta $\boldsymbol{k}'$ and $\boldsymbol{p}$ inside the resonance ring and $\boldsymbol{p}'$ and $\boldsymbol{k}$ outside the resonance ring. To account for processes where $(\boldsymbol{k}', \boldsymbol{p})$ and $(\boldsymbol{p}', \boldsymbol{k})$ are exchanged, we multiply the rate by 2. We consider the process for which $\Delta\varepsilon_1 = \varepsilon_{\boldsymbol{k}'-} - \varepsilon_{\boldsymbol{k}+} < 0$, and $\Delta\varepsilon_2 = \varepsilon_{\boldsymbol{p}'-} - \varepsilon_{\boldsymbol{p}+} > 0$. The amplitude of such a process is suppressed by a factor of $(V/\Omega)^2$, see Supplementary Fig. 2c. We also assume that the amplitude of the process where $\Delta\varepsilon_1 > 0$, and $\Delta\varepsilon_2 < 0$ constructively interfere with the previous case such that the matrix element for both processes is twice the matrix element of each one of them. Following from the above, we approximate $\mathcal{P}^{(1)}_{\rm ee} \approx 2(2UV/a^2\Omega)^2$. Notice that there is an additional scenario included in $io \to io$ processes, corresponding to $|\boldsymbol{k}|, |\boldsymbol{k}'| < k_{\rm R}$ and $|\boldsymbol{p}|, |\boldsymbol{p}'| > k_{\rm R}$. This scenario gives much smaller contribution to the rate since it includes transitions between almost orthogonal pseudospin states. We thus neglect it in our analysis.

To evaluate the integral in Supplementary Eq. (61) we introduce a new variable, ω, which splits the energy-conservation δ-function as follows, $\delta(\Delta\varepsilon_1 + \Delta\varepsilon_2 + \Omega) = \int d\omega \delta(\Delta\varepsilon_3 + \omega)\delta(\Delta\varepsilon_4 + \Omega - \omega)$. Here, we defined $\Delta\varepsilon_3 \equiv \varepsilon_{\boldsymbol{k}'-} - \varepsilon_{\boldsymbol{p}+}$ and $\Delta\varepsilon_4 \equiv \varepsilon_{\boldsymbol{p}'-} - \varepsilon_{\boldsymbol{k}+}$, such that $\Delta\varepsilon_1 + \Delta\varepsilon_2 = \Delta\varepsilon_3 + \Delta\varepsilon_4$. Therefore, ω denotes the energy transfer inside the resonance ring and $\Omega - \omega$ the energy transfer outside the resonance ring (see Supplementary Fig. 4b). It is also useful to define the momentum transfer inside the resonance ring, $\boldsymbol{q}' = \boldsymbol{p} - \boldsymbol{k}'$. By conservation of the total momentum, it equals the momentum transfer outside the resonance ring, $\boldsymbol{q}' = \boldsymbol{p}' - \boldsymbol{k}$. The bandstructure near the Γ-point restricts the values of ω and $|\boldsymbol{q}'|$ to $\omega < \delta E$ and $|\boldsymbol{q}'| < 2k_{\rm R}$. Therefore, given the constraint $-\Delta\varepsilon_4 = \Omega - \omega$, we can approximate $\Delta\varepsilon_4 \approx 2\varepsilon_{\boldsymbol{p}'-} \approx -2\varepsilon_{\boldsymbol{k}+}$, since $\delta E \ll \Omega$.

We use the last approximation to replace $\Delta\varepsilon_4$ by $-2\varepsilon_{\boldsymbol{k}+}$ in the energy-conservation δ-function in Supplementary Eq. (61), and integrate over $\boldsymbol{k}$, yielding $\int d^2\boldsymbol{k}\delta(\Delta\varepsilon_4 + \Omega - \omega) \approx \pi m_*$. Remarkably, due to the nearly parabolic dispersion outside the resonance ring with a density of states which is constant as a function of energy, the result of the $\boldsymbol{k}$-integral is independent of $\Omega - \omega$. Next, we perform the ω-integral over $\delta(\Delta\varepsilon_3 + \omega)$, yielding 1. Finally, we perform the trivial $\boldsymbol{p}$ and $\boldsymbol{k}'$-integrals over a constant function remained from the previous integrals, yielding a factor of $\mathcal{A}_{\rm R}$ for each of the variables. Collecting all the factors, we arrive at

$$\Gamma_{\rm ee} \approx \frac{\mathcal{A}_{\rm R}^2 U^2 m_*}{2\pi^4}\left(\frac{V}{\Omega}\right)^2. \qquad (62)$$

e. Phonon-assisted interband recombination rate

Here, we discuss phonon-assisted interband relaxation processes. Interband relaxation serves as a sink mechanism for the excitations created by the heating processes (described in Supplementary Note 3 d). Electrons transferred to the UFB by the heating processes first undergo fast intraband relaxation followed by multiple-phonon emission, transferring electrons to the bottom of the UFB. Then, they recombine with the holes in the LFB, which were, in turn, transferred to the top of the UFB by similar processes. Here we focus on such interband electron-hole recombination processes predominantly mediated by phonons (see Supplementary Fig. 3d). We approximate the states involved in the scattering by the RWA states at the resonance ring, see Supplementary Eq. (10).

We expect the ROC of the density due to interband-recombination to be proportional to the densities of the electrons and holes in the UFB and LFB respectively. We thus define $\dot{n}_{\mathrm{e}}|_{\mathrm{inter}} = -\Lambda_{\mathrm{inter}} n_{\mathrm{e}} n_{\mathrm{h}}$, where

$$\Lambda_{\mathrm{inter}} = -\frac{1}{n_{\mathrm{e}} n_{\mathrm{h}}} \int \frac{a^2 d^2\boldsymbol{k} d^2\boldsymbol{k}'}{(2\pi)^4} (\dot{f}_{\boldsymbol{k}+})_{\mathrm{s},\boldsymbol{k}'-}. \tag{63}$$

Here $(\dot{f}_{\boldsymbol{k}+})_{\mathrm{s},\boldsymbol{k}'-}$ is given by Eq. (46) where we take only the $l = 0$ term and estimate the energy transfer by $\sim \Delta_{\mathrm{F}}$, leading to

$$(\dot{f}_{\boldsymbol{k},+})_{\mathrm{s},\boldsymbol{k}'-} \approx -2\pi \mathcal{P}_{\mathrm{s}}^{(0)}(\boldsymbol{k}+,\boldsymbol{k}'-) \rho_{\mathrm{s}}(\boldsymbol{q},\Delta_{\mathrm{F}}) F(\boldsymbol{k}+,\boldsymbol{k}'-). \tag{64}$$

The function $F(\boldsymbol{k}+,\boldsymbol{k}'-)$ is non zero only when $|\boldsymbol{k}|, |\boldsymbol{k}'| \approx k_{\mathrm{R}}$. Therefore, we estimate $|\boldsymbol{q}| = |\boldsymbol{k}' - \boldsymbol{k}| \approx 2k_{\mathrm{R}} |\sin(\Delta\theta/2)|$, where $\Delta\theta = \theta - \theta'$, and θ, θ' are the angles of the vectors $\boldsymbol{k}$ and $\boldsymbol{k}'$ with respect to the $\hat{\boldsymbol{x}}$ axis. We evaluate $\mathcal{P}_{\mathrm{s}}^{(0)}$ by the matrix elements of the electron-phonon coupling [Supplementary Eq. (48)] estimated at $\omega = \Delta_{\mathrm{F}}$ with Floquet states given in Supplementary Eq. (10), leading to $\mathcal{P}_{\mathrm{s}}^{(0)} \approx (4g_{\mathrm{s}}^2 k_{\mathrm{R}}^2/\Delta_{\mathrm{F}}) \sin^4(\Delta\theta/2)$. In the analysis we consider the limit, $\Delta_{\mathrm{F}} \gg 2v_{\mathrm{s}} k_{\mathrm{R}}$, which allows us to treat the density of states of the phonons as constant, $\rho_{\mathrm{s}}(\boldsymbol{q},\Delta_{\mathrm{F}}) \approx \rho_{\mathrm{s}}^0$.

Now, we turn to the evaluation of the integral in Supplementary Eq. (63). The approximate form of $(\dot{f}_{\boldsymbol{k}+})_{\mathrm{s},\boldsymbol{k}'-}$, discussed in Supplementary Eq. (64) and further approximations below it, lead to a separation of the integrand into a product of radial and angular terms of $\boldsymbol{k}$ and $\boldsymbol{k}'$. We thus transform $d^2\boldsymbol{k} \to d\theta k dk$ and $d^2\boldsymbol{k}' \to d\theta' k' dk'$. We first integrate over the the radial components, which only appear in $F(\boldsymbol{k}+,\boldsymbol{k}'-)$, leading to $\frac{1}{(2\pi)^2}\int k dk k' dk' F(\boldsymbol{k}+,\boldsymbol{k}'-) = n_{\mathrm{e}} n_{\mathrm{h}}$. Then we integrate over the rest of the components, which have only angular dependencies, proportional to $\sin^4(\Delta\theta/2)$. We thus arrive at the following expression,

$$\Lambda_{\mathrm{inter}} \approx \frac{3\pi g_{\mathrm{s}}^2 \rho_{\mathrm{s}}^0 a^2 k_{\mathrm{R}}^2}{\Delta_{\mathrm{F}}}. \tag{65}$$

f. Phonon-assisted intraband relaxation rate

Next, we estimate the phonon-assisted intraband relaxation rate. Phonon-assisted intraband relaxation is the dominant mechanism for reducing the effective temperature of the excitations created by heating processes (described in Supplementary Note 3 d) within each of the bands. Namely, it transfers electrons in the UFB to the bottom of the band and holes in the LFB to the top of the band. To account for the relaxation rate, we divide the population of electrons into two subsets occupying high and low-quasienergy states of electron densities $n_{\mathrm{e}}^{\mathrm{H}}$ and $n_{\mathrm{e}}^{\mathrm{L}}$, respectively [cf. Supplementary Eqs. (39) and (42)]. For concreteness, in what follows we consider an EFM phase, associated with a sharp Fermi surface for the distribution of the electrons (the analysis in the EFI phase is similar). In this case, the separation between high- and low-quasienergy subsets for the distribution of the electrons is at the effective chemical potential measured relative to the UFB bottom, μ_{e} [see an extended discussion in Supplementary Note 3 b and Supplementary Fig. 2a for an illustration].

For the EFM phase, intraband relaxation in the UFB is essentially electron-hole pair annihilation across the effective chemical potential with the rate $\dot{n}_{\mathrm{e}}^{\mathrm{L}}|_{\mathrm{intra}} = \Lambda_{\mathrm{intra}} n_{\mathrm{e}}^{\mathrm{H}} \bar{n}_{\mathrm{e}}^{\mathrm{L}}$, where $\bar{n}_{\mathrm{e}}^{\mathrm{L}}$ is the density of unoccupied states in the low-quasienergy subset. The rate Λ_{intra} is given by

$$\Lambda_{\mathrm{intra}} = \frac{1}{n_{\mathrm{e}}^{\mathrm{H}} \bar{n}_{\mathrm{e}}^{\mathrm{L}}} \int \frac{a^2 d^2\boldsymbol{k} d^2\boldsymbol{k}'}{(2\pi)^4} (\dot{f}_{\boldsymbol{k}+})_{\mathrm{s},\boldsymbol{k}'+}, \tag{66}$$

where the range of $\boldsymbol{k}'$-integral is all the states in the high-energy sector, corresponding to $\varepsilon_{\boldsymbol{k}'+} > \Delta_{\mathrm{F}}/2 + \mu_{\mathrm{e}}$. Subsequently, the range of $\boldsymbol{k}$-integral is all the states in the low-quasienergy sector, $\Delta_{\mathrm{F}}/2 < \varepsilon_{\boldsymbol{k}+} < \Delta_{\mathrm{F}}/2 + \mu_{\mathrm{e}}$. We estimate $(\dot{f}_{\boldsymbol{k}+})_{\mathrm{s},\boldsymbol{k}'+}$ [given in Supplementary Eq. (46)] by the $l = 0$ term, thus this is not a Floquet-Umklapp process. The typical energy transfer in these processes equals the effective temperature of the electrons $\sim T_{\mathrm{e}}$. Therefore, we approximate

$$(\dot{f}_{\boldsymbol{k}+}) \approx 2\pi \mathcal{P}_{\mathrm{s}}^{(0)}(\boldsymbol{k}+,\boldsymbol{k}'+) \rho_{\mathrm{s}}(\boldsymbol{q},T_{\mathrm{e}}) F(\boldsymbol{k}+,\boldsymbol{k}'+). \tag{67}$$

Since both of the momenta involved are close to the band minimum at $|\boldsymbol{k}|, |\boldsymbol{k}'| = k_{\mathrm{R}}$, the momentum transfer is given by $|\boldsymbol{q}| \approx 2k_{\mathrm{R}} |\sin(\Delta\theta/2)|$, where $\Delta\theta$ is the angle between the vectors $\boldsymbol{k}$ and $\boldsymbol{k}'$. For low temperatures $T_{\mathrm{e}} \ll v_{\mathrm{s}} k_{\mathrm{R}}$, the density of states $\rho_{\mathrm{s}}(\boldsymbol{q},T_{\mathrm{e}})$, restricts the momentum transfers within a small circle in the momentum space, $|\boldsymbol{q}| < T_{\mathrm{e}}/v_{\mathrm{s}}$, illustrated by a red circle in Supplementary Fig. 3e. To further simplify the expression in Supplementary Eq. (67), we use the paraxial approximation of small angles, $|\boldsymbol{q}| \approx k_{\mathrm{R}} \Delta\theta$. We estimate $\mathcal{P}_{\mathrm{s}}^{(0)}$ by the matrix elements of the electron-phonon coupling [Supplementary Eq. (48)] estimated at $\omega = T_{\mathrm{e}}$, using the Floquet states given in Supplementary Eq. (10). The above approximations result in $\mathcal{P}_{\mathrm{s}}^{(0)} \approx g_{\mathrm{s}}^2 k_{\mathrm{R}}^2 \Delta\theta^2/T_{\mathrm{e}}$, and

$\rho_\mathrm{s}(\boldsymbol{q},T_\mathrm{e}) \approx \rho_\mathrm{s}^0 T_\mathrm{e}/\sqrt{T_\mathrm{e}^2-(v_\mathrm{s}k_\mathrm{R}\Delta\theta)^2}$, where $|\Delta\theta| < \theta_\mathrm{s}$; $\theta_\mathrm{s} = T_\mathrm{e}/(k_\mathrm{R}v_\mathrm{s})$.

Now, we are ready to evaluate the integral in Supplementary Eq. (66); we will use polar coordinates. The integrand splits to a product of terms dependent on the radial and angular components of the momentum. The integral over the radial part yields $\frac{1}{(2\pi)^2}\int kdkk'dk'F(\boldsymbol{k}+,\boldsymbol{k}'+) = n_\mathrm{e}^\mathrm{H}\bar{n}_\mathrm{e}^\mathrm{L}$. In turn, the angular part yields $\int_{-\theta_\mathrm{s}}^{\theta_\mathrm{s}} d\Delta\theta\Delta\theta^2/\sqrt{1-(\Delta\theta/\theta_\mathrm{s})^2} = \pi\theta_\mathrm{s}^3/2$. Combining the above results of the angular and radial integrals, we arrive at

$$\Lambda_\mathrm{intra} \approx \frac{\pi g_\mathrm{s}^2\rho_\mathrm{s}^0 a^2 T_\mathrm{e}^2}{2k_\mathrm{R}v_\mathrm{s}^3}. \tag{68}$$

A similar analysis for the EFI phase yields the same estimate [Supplementary Eq. (68)]. Subsequently, an analysis for holes gives the same result upon a replacement $T_\mathrm{e} \to T_\mathrm{h}$ (due to the particle-hole symmetry of the Floquet bands).

g. Equilibration of the electron and hole distributions via electron-electron scattering

Here we estimate the rate of intraband thermalization processes induced by electron-electron scattering (depicted in Supplementary Fig. 3f). We consider scattering processes in which one of the incoming electrons is in the UFB, and one in the LFB, and likewise for the outgoing electrons. Thus, no interband particle transfer is involved. These processes tend to equalize the temperatures of the electron and hole populations as a result of the collisions between them. We do not consider intraband scattering, where both electrons are in the UFB or LFB, as such processes do not contribute if the distribution within the band can be described by a Fermi function (i.e., in a phase-space-local equilibrium). For simplicity, we consider a situation where the electron and hole populations in the two bands have close temperatures, such that the difference between the temperatures, $\Delta T = T_\mathrm{e} - T_\mathrm{h}$, is much smaller than the mean temperature, $T = (T_\mathrm{e}+T_\mathrm{h})/2$, $|\Delta T| \ll T$.

We define the thermalization rate by $\dot{n}_\mathrm{e}^\mathrm{L}|_\mathrm{ee} = \gamma_\mathrm{ee}\Delta T n_\mathrm{e}^\mathrm{H}\bar{n}_\mathrm{e}^\mathrm{L} n_\mathrm{h}^\mathrm{H}\bar{n}_\mathrm{h}^\mathrm{L}$, see the discussion above Supplementary Eq. (44), where

$$\gamma_\mathrm{ee} = \frac{\int a^4 d^2\boldsymbol{k}d^2\boldsymbol{k}'d^2\boldsymbol{p}d^2\boldsymbol{p}'(\dot{f}_{\boldsymbol{k}+})_\mathrm{ee}\delta^{(2)}(\boldsymbol{k}+\boldsymbol{p}-\boldsymbol{k}'-\boldsymbol{p}')}{(2\pi)^6\Delta T n_\mathrm{e}^\mathrm{H}\bar{n}_\mathrm{e}^\mathrm{L} n_\mathrm{h}^\mathrm{H}\bar{n}_\mathrm{h}^\mathrm{L}}. \tag{69}$$

Here $(\dot{f}_{\boldsymbol{k}+})_\mathrm{ee}$ is given by Supplementary Eq. (50), where we take only $l=0$. We assume that $\boldsymbol{k}$ and $\boldsymbol{k}'$ describe states in the UFB and $\boldsymbol{p}$ and $\boldsymbol{p}'$ in the LFB, i.e., $(\nu,\nu',\mu,\mu') = (+,+,-,-)$, according to the notations in Supplementary Eq. (50). For concreteness, we consider an EFM phase for electrons in the UFB (the analysis for the EFI phase is similar). The holes in the LFB form a non-degenerate distribution. The range of $\boldsymbol{k}'$-integral is all the states in the high-energy sector of the UFB, corresponding to $\varepsilon_{\boldsymbol{k}'+} > \Delta_\mathrm{F}/2+\mu_\mathrm{e}$. Subsequently, the range of $\boldsymbol{k}$-integral is all the states in the low-quasienergy sector of the UFB, $\Delta_\mathrm{F}/2 < \varepsilon_{\boldsymbol{k}+} < \Delta_\mathrm{F}/2+\mu_\mathrm{e}$. The $\boldsymbol{p}$ and $\boldsymbol{p}'$-integrals are in the range $-\Delta_\mathrm{F}/2-T < \varepsilon_{\boldsymbol{p}-} < -\Delta_\mathrm{F}/2$ and $\varepsilon_{\boldsymbol{p}'-} < -\Delta_\mathrm{F}/2-T$, according to the definition of the low and high-quasienergy sectors in the LFB, see the text surrounding Supplementary Eq. (42) and Supplementary Fig. 2.

In the limit $\Delta T/T \to 0$, we approximate $(\dot{f}_{\boldsymbol{k}+})_\mathrm{ee}$ by the leading (linear) order in ΔT such that γ_ee is a constant in ΔT by the definition [Supplementary Eq. (69)]. To extract the first-order term in ΔT from $(\dot{f}_{\boldsymbol{k}+})_\mathrm{ee}$, we describe the electron and hole distributions as Fermi functions and use this description in the expression for $F_\mathrm{ee}(\boldsymbol{k}+,\boldsymbol{p}-;\boldsymbol{k}'+\boldsymbol{p}'-)$ [given in Supplementary Eq. (51)]. In particular, we use the property $1-\frac{1}{e^x+1} = e^x\left(\frac{1}{e^x+1}\right)$ to switch between f and $\bar{f}$-terms in Supplementary Eq. (51), leading to

$$F_\mathrm{ee} = (1-e^{\Delta\varepsilon_1/T_\mathrm{e}}e^{\Delta\varepsilon_2/T_\mathrm{h}})F(\boldsymbol{k}+,\boldsymbol{k}'+)F(\boldsymbol{p}-,\boldsymbol{p}'-), \tag{70}$$

where $F(\boldsymbol{k}\nu,\boldsymbol{k}'\nu')$ is defined in Supplementary Eq. (47). The term in the brackets in the RHS of Supplementary Eq. (70) is linear to the leading order in ΔT, i.e., $1-e^{\Delta\varepsilon_1/T_\mathrm{e}}e^{\Delta\varepsilon_2/T_\mathrm{h}} = \Delta\varepsilon_1\Delta T/T^2 + \mathcal{O}(\Delta T^2)$ (since by energy conservation $\Delta\varepsilon_1+\Delta\varepsilon_2 = 0$). We thus take only the zeroth order in ΔT of the rest of the terms in the RHS, to arrive at

$$F_\mathrm{ee}(\boldsymbol{k}+,\boldsymbol{p}-;\boldsymbol{k}'+\boldsymbol{p}'-) \approx \frac{\Delta\varepsilon_1\Delta T}{T^2}F(\boldsymbol{k}+,\boldsymbol{k}'+)F(\boldsymbol{p}-,\boldsymbol{p}'-), \tag{71}$$

where all the Fermi functions in F are evaluated at $\Delta T = 0$.

Next, we evaluate $\mathcal{P}_\mathrm{ee}^{(0)}$, employing Supplementary Eq. (52) and Floquet states given in Supplementary Eq. (10), which leads to

$$\begin{aligned}&\mathcal{P}_\mathrm{ee}^{(0)}(\boldsymbol{k}+,\boldsymbol{p}-;\boldsymbol{k}'+,\boldsymbol{p}'-) \approx\\ &\approx (U/a^2)^2\cos^2[(\theta-\phi)/2]\cos^2[(\theta'-\phi')/2].\end{aligned} \tag{72}$$

Here, we used polar coordinates, $\boldsymbol{k} = (k\sin(\theta),k\cos(\theta))$, $\boldsymbol{k}' = (k'\sin(\theta'),k'\cos(\theta'))$, $\boldsymbol{p} = (p\sin(\phi),p\cos(\phi))$, and $\boldsymbol{p}' = (p'\sin(\phi'),p'\cos(\phi'))$.

Once we estimated all the ingredients appearing in the definition of $(\dot{f}_{\boldsymbol{k}+})_\mathrm{ee}$ [Supplementary Eq. (50)], we are in the position to evaluate the integral in Supplementary Eq. (69). To this end, we first replace each of the two-dimensional momenta integrals by the energy and angle integrals using polar representation. For example, $\int\frac{d^2\boldsymbol{k}}{(2\pi)^2} = \int d\varepsilon_k D_\mathrm{F}(\varepsilon_k)\int\frac{d\theta}{2\pi}$, where $D_\mathrm{F}(\varepsilon_k)$ is the density of states [given in Supplementary Eq. (23)].

We start with the angular part of the integral. We fix two of the angles, θ and θ', and find the other two, ϕ and ϕ' by momentum conservation. If the scattering is exactly at the resonance ring, i.e., $k = k' = p = p' = k_\mathrm{R}$, conservation of the total momentum implies either (i)

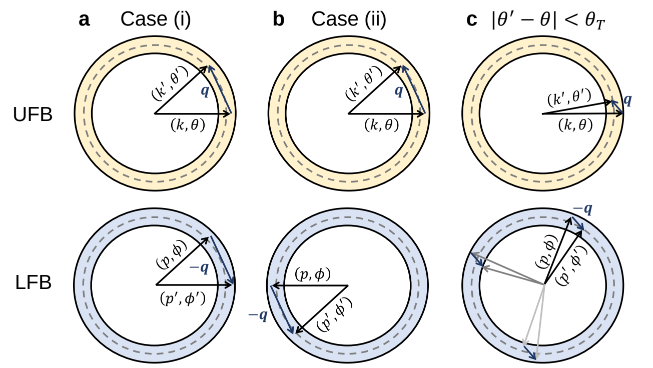

Supplementary Figure 5. **Electron-electron scattering processes leading to equilibration of the electron and hole distributions.** Yellow (top) and blue (bottom) rings indicate the area of states in the reciprocal space available for scattering near the resonance ring (gray dashed line), the width of the ring is approximately $\sim k_{\mathrm{R}}\theta_T$. The black arrows in the upper circle represent the momentum of the electron in the UFB before and after the scattering event, with a momentum transfer $\boldsymbol{q}$ indicated by blue arrow. Similarly, the black arrows in the figure at the bottom correspond to momentum of an electron in the LFB before and after the transition, with a momentum transfer $-\boldsymbol{q}$, due to total momentum conservation. **a** Possible scattering event for the case (i), for which $\phi \approx \theta'$ and $\phi' \approx \theta$. **b** Possible scattering event for the case (ii), for which $\phi \approx \pi+\theta$ and $\phi' \approx \pi+\theta'$. This case is Pauli suppressed as it scatters between almost orthogonal pseudospin states [cf. Supplementary Eq. (72)]. **c** Possible scattering events when $|\theta'-\theta| < \theta_T$. In this case, there are many possible configurations of the angle ϕ', all satisfying momentum conservation.

$\phi = \theta'$ and $\phi' = \theta$, or (ii) $\phi = \pi + \theta$ and $\phi' = \pi + \theta'$, see Supplementary Fig. 5a and b. In practice, the momentum amplitude fluctuates around k_{R}, due to the thermal and doping-induced width of the distributions, leading to fluctuations of the angles, $\phi = \phi_0 + \delta\phi$ and $\phi' = \phi_0' + \delta\phi'$, where ϕ_0 and ϕ_0' are the solutions for ϕ and ϕ' in terms of θ and θ' which we found in the cases (i) and (ii) above. To find how $\delta\phi$ and $\delta\phi'$ depend on the fluctuations in the amplitudes (defined as $\delta k = k - k_{\mathrm{R}}$ and similarly for other momenta), we expand the conservation of momentum in the $\hat{\boldsymbol{x}}$ and $\hat{\boldsymbol{y}}$ directions to the first order in the fluctuations. The linearized equations for the case (i) read

$$\delta\phi k_{\mathrm{R}} \sin(\theta') = \delta k - \delta p' + (\delta p - \delta k')\cos(\theta') \quad (73a)$$

$$\delta\phi k_{\mathrm{R}} \cos(\theta') = k_{\mathrm{R}}\delta\phi' + (\delta k' - \delta p)\sin(\theta'), \quad (73b)$$

where we set $\theta = 0$, as the system is rotational symmetric. The momentum-conserving δ-function for the case (i) then reads

$$\delta_1^{(2)}(\boldsymbol{k}+\boldsymbol{p}-\boldsymbol{k}'-\boldsymbol{p}') = \frac{\delta(\phi-\theta'-\delta\phi_1)\delta(\phi'-\theta-\delta\phi_1')}{k_{\mathrm{R}}^2|\sin(\theta')|}, \quad (74)$$

where $\delta\phi_1$ and $\delta\phi_1'$ obtained from the solution to Supplementary Eq. (73) for $\delta\phi$ and $\delta\phi'$ as functions of $\delta k, \delta k', \delta p, \delta p'$ and θ'. Next, we consider the case (ii). Here, the momentum conserving δ-functions reads

$$\delta_2^{(2)}(\boldsymbol{k}+\boldsymbol{p}-\boldsymbol{k}'-\boldsymbol{p}') = \\ = \frac{\delta(\phi-\pi-\theta-\delta\phi_2)\delta(\phi'-\pi-\theta'-\delta\phi_2')}{k_{\mathrm{R}}^2|\sin(\theta')|}. \quad (75)$$

In the case (ii), $\delta\phi_2$ and $\delta\phi_2'$ are obtained from equations similar to Supplementary Eq. (73) with an exchange $\delta p \leftrightarrow \delta p'$, $\delta\phi \leftrightarrow \delta\phi'$. The full momentum conserving δ-function is a sum of Supplementary Eqs. (74) and (75).

The cases (i) and (ii) above, apply when $|\boldsymbol{k}'-\boldsymbol{k}| \gg \delta k, \delta k', \delta p, \delta p'$. We introduce a cutoff parameter, θ_T such that for $|\theta'| > \theta_T$, the momentum conservation is ensured by the sum of Supplementary Eqs. (74) and (75). (Recall that $|\boldsymbol{k}'-\boldsymbol{k}| \approx k_{\mathrm{R}}|\theta'|$ for $\theta = 0$ and small θ'.) This cutoff depends on the average values of $\delta k, \delta k', \delta p$ and $\delta p'$, therefore it is in general a function of T and μ_{e}. However as we shall show later, the result only weakly (logarithmically) depends on the cutoff, therefore its T and μ_{e} dependence is not significant. For $|\theta'| < \theta_T$, the momentum can be conserved for many values of ϕ', see Supplementary Fig. 5c. In this case, we fix ϕ' and $\theta = 0$ and find the corresponding angles ϕ and θ' from the momentum conservation. Defining $\phi = \phi' + \delta\phi$, where $\delta\phi$ is small (of the order of θ'), we arrive at the equations for $\delta\phi$ and θ',

$$\delta\phi k_{\mathrm{R}} \sin(\phi') = \delta k - \delta k' + (\delta p - \delta p')\cos(\phi') \quad (76a)$$

$$\delta\phi k_{\mathrm{R}} \cos(\phi') = k_{\mathrm{R}}\theta' + (\delta p' - \delta p)\sin(\phi'). \quad (76b)$$

The momentum conserving δ-function then reads

$$\delta_3^{(2)}(\boldsymbol{k}+\boldsymbol{p}-\boldsymbol{k}'-\boldsymbol{p}') = \frac{\delta(\phi-\phi'-\delta\phi_3)\delta(\theta'-\theta_3')}{k_{\mathrm{R}}^2|\sin(\phi')|}. \quad (77)$$

where $\delta\phi_3$ and θ_3' are the solutions to Supplementary Eqs. (76). Supplementary Eq. (77) is valid above approximately same cutoff $|\phi'| > \theta_T$.

We are now ready to perform the angular integral in Supplementary Eq. (69). The only angle-dependent parts in this integral are the momentum-conserving δ-functions [Supplementary Eqs. (74), (75) and (77)] and $\mathcal{P}_{\mathrm{ee}}^{(0)}$ given in Supplementary Eq. (72). We define an angular integral over each of the δ-functions, $I_{\mathrm{ang}} = I_{\mathrm{ang}}^{(1)} + I_{\mathrm{ang}}^{(2)} + I_{\mathrm{ang}}^{(3)}$, where $I_{\mathrm{ang}}^{(1,2)} = \int_{|\theta'|>\theta_T} \frac{d\theta d\theta' d\phi d\phi'}{(2\pi)^4}\delta_{1,2}^{(2)}\mathcal{P}_{\mathrm{ee}}^{(0)}$, and $I_{\mathrm{ang}}^{(3)} = \int_{|\theta'|<\theta_T, |\phi'|>\theta_T} \frac{d\theta d\theta' d\phi d\phi'}{(2\pi)^4}\delta_3^{(2)}\mathcal{P}_{\mathrm{ee}}^{(0)}$. The integral over θ can be trivially performed in each of $I_{\mathrm{ang}}^{(i)}$, yielding 2π. Next, we perform the integrals over the δ-functions, leading to

$$I_{\mathrm{ang}}^{(1)} = \frac{U^2}{a^4}\int \frac{d\theta'}{(2\pi)^3} \frac{\cos^2(\frac{\theta'+\delta\phi_1}{2})\cos^2(\frac{\theta'-\delta\phi_1'}{2})}{k_{\mathrm{R}}^2|\sin(\theta')|} \quad (78)$$

$$I_{\mathrm{ang}}^{(2)} = \frac{U^2}{a^4}\int \frac{d\theta'}{(2\pi)^3} \frac{\sin^2(\frac{\delta\phi_2}{2})\sin^2(\frac{\delta\phi_2'}{2})}{k_{\mathrm{R}}^2|\sin(\theta')|} \quad (79)$$

$$I^{(3)}_{\rm ang} = \frac{U^2}{a^4}\int \frac{d\phi'}{(2\pi)^3}\frac{\cos^2(\frac{\phi'+\delta\phi_3}{2})\cos^2(\frac{\theta'_3-\phi'}{2})}{k_{\rm R}^2|\sin(\phi')|} \tag{80}$$

The θ'-integral in Supplementary Eqs. (78) and (79) and ϕ'-integral in Supplementary Eq. (80) are in the range $[\theta_T, 2\pi-\theta_T]$.

The last integral [Supplementary Eq. (80)] is nonzero when $|\theta'_3| < \theta_T$ [recall that θ'_3 is obtained from Supplementary Eqs. (76)]. We choose θ_T to be large enough for this condition to be satisfied for any allowed value of $\delta k, \delta k', \delta p, \delta p', \delta\phi$ and ϕ'. Choosing too large value of θ_T will lead to an underestimate for the total rate, as some of the values of θ' will not be included in the calculation.

Turning back to Supplementary Eqs. (78)-(80), we neglect small angles $\delta\phi_1$ and $\delta\phi'_1$ with respect to θ' in Supplementary Eq. (78), and $\delta\phi_3$ and θ'_3 with respect to ϕ' in Supplementary Eq. (80). We also neglect $I^{(2)}_{\rm ang}$ as its value is proportional to the squares of small angles $\propto \delta\phi_2^2\delta\phi_2'^2$. Therefore, we arrive at $I_{\rm ang} \approx \frac{2U^2}{(2\pi)^3a^4k_{\rm R}^2}\int d\theta' \frac{\cos^4(\theta'/2)}{|\sin(\theta')|}$, where $\theta' \in [\theta_{\rm T}, 2\pi-\theta_{\rm T}]$. This results, in the limit of small $\theta_{\rm T}$, in $I_{\rm ang} \approx U^2\frac{2\log(2/\theta_{\rm T})-1}{(2\pi)^3a^4k_{\rm R}^2}$.

We note that in the calculation of the angular integral we omitted parameter regimes in which the angular integral can not be separated from the amplitude integral, by introducing the cutoff θ_T. As the angular phase space corresponding to this regime is small, its omission only weakly affects the final result.

Now, we turn to the integration over the energy-dependent terms arising from $F_{\rm ee}$ [Supplementary Eq. (71)] and $\delta(\Delta\varepsilon_1+\Delta\varepsilon_2)$. Recall, that we consider an EFM phase for the electrons in the UFB, and describe the distribution of holes by Supplementary Eq. (29). We define $I_{\rm en}$ as the energy integral over the energy-dependent terms, given by

$$\begin{aligned} I_{\rm en} = &\int_0^{\mu_{\rm e}} d\varepsilon_k \int_{\mu_{\rm e}}^{\infty} d\varepsilon_{k'} \int_{-T}^{0} d\varepsilon_p \int_{-\infty}^{-T} d\varepsilon_{p'} \\ &D_{\rm F}(\varepsilon_k)D_{\rm F}(\varepsilon_{k'})D_{\rm F}(-\varepsilon_p)D_{\rm F}(-\varepsilon_{p'})\frac{\Delta T(\varepsilon_{k'}-\varepsilon_k)}{T^2} \\ &\delta(\varepsilon_{k'}-\varepsilon_k+\varepsilon_{p'}-\varepsilon_p)f_{\varepsilon_{k'}+}\bar{f}_{\varepsilon_k+}f_{\varepsilon_{p'}-}\bar{f}_{\varepsilon_p-}, \end{aligned} \tag{81}$$

where ε_k and $\varepsilon_{k'}$ are accounted from the bottom of the UFB and ε_p and $\varepsilon_{p'}$ are accounted from the top of the LFB (and are negative). Assuming the electrons in the UFB are deep in the EFM regime, $\mu_{\rm e}/T \gg 1$, we approximate $D_{\rm F}(\varepsilon_k)$ and $D_{\rm F}(\varepsilon_{k'})$ by $D_{\rm F}(\mu_{\rm e})$ and take the lower limit of the ε_k-integral to $-\infty$. We set $\bar{f}_{\varepsilon_p-} \approx z_{\rm h}e^{\varepsilon_p/T}$ and $f_{\varepsilon_{p'}-} \approx 1$, assuming $z_h \ll 1$, while $\bar{f}_{\varepsilon_k+} = \frac{1}{e^{-(\varepsilon_k-\mu_{\rm e})/T}+1}$ and $f_{\varepsilon_{k'}+} = \frac{1}{e^{(\varepsilon_{k'}-\mu_{\rm e})/T}+1}$. To evaluate the integral, we shift ε_k and $\varepsilon_{k'}$ by $\mu_{\rm e}$ and rescale all the energies by T, $\varepsilon_k = \mu_{\rm e}-Tx$, $\varepsilon_{k'} = \mu_{\rm e}+Ty$, $\varepsilon_p = -Tz$, and $\varepsilon_{p'} = -Tw$, leading to

$$I_{\rm en} = C_1C_2\frac{\Delta T}{T^2}n_{\rm e}^{\rm H}\bar{n}_{\rm e}^{\rm L}n_{\rm h}^{\rm H}\bar{n}_{\rm h}^{\rm L}, \tag{82}$$

where

$$\begin{aligned} C_1 = &\int_0^\infty dx\int_0^\infty dy\int_0^1 dz\int_1^\infty dw\frac{y+x}{\sqrt{zw}}\times \\ &\times\delta(y+x+z-w)\frac{1}{e^x+1}\frac{1}{e^y+1}e^{-z} \approx 0.94, \end{aligned} \tag{83}$$

and $C_2 = [2\sqrt{\pi}{\rm erfc}(1)\log^2(2)]^{-1} \approx 3.73$. In this calculation we used explicit expressions of the densities of electron and hole subpopulations given in Supplementary Eqs. (40) and (43).

Combining the angular and energy integrals, we arrive at $\gamma_{\rm ee} = \frac{(2\pi)^3a^4I_{\rm ang}I_{\rm en}}{\Delta T n_{\rm e}^{\rm H}\bar{n}_{\rm e}^{\rm L}n_{\rm h}^{\rm H}\bar{n}_{\rm h}^{\rm L}}$ leading to

$$\gamma_{\rm ee} \approx \frac{C_\gamma U^2}{k_{\rm R}^2T^2}, \tag{84}$$

where $C_\gamma = [2\log(2/\theta_{\rm T})-1]C_1C_2$. The same calculation for the electrons in the EFI phase and for the holes, leads to a similar expression for $\gamma_{\rm ee}$, up to a numerical factor $\mathcal{O}(1)$.

h. Solution of the extended rate equations

Now, we are ready to solve Supplementary Eqs. (44) and (45). Our goal is to find the dependence of the effective temperatures and chemical potentials of the electrons and holes on the speed of sound, $v_{\rm s}$, the doping, Δn and the "balance parameter", κ [defined in Supplementary Eq. (37)].

1. *Electron and hole temperatures for* $\gamma_{\rm ee} = 0$

First, we assume $\gamma_{\rm ee} = 0$, which corresponds to the model used for the full numerical simulation of the kinetic equation. Later, we show that if we include a finite $\gamma_{\rm ee}$ in the model, the distribution of the electrons, whose density is significantly larger then the density of the holes, will be almost unaffected. As a result, we expect the predicted phase boundary in Fig. 3 in the main text, to remain almost unchanged in the presence of electron-electron scattering processes which equilibrate the electron and hole populations. For $\gamma_{\rm ee} = 0$, each temperature, $T_{\rm e}$ or $T_{\rm h}$, is obtained from its respective equation, Supplementary Eq. (44) or Supplementary Eq. (45).

In what follows, we obtain $T_{\rm e}$ from the solution to Supplementary Eq. (44), which for $\gamma_{\rm ee} = 0$ reads

$$\Lambda_{\rm intra}(T_{\rm e})n_{\rm e}^{\rm H}\bar{n}_{\rm e}^{\rm L} = \Lambda_{\rm inter}n_{\rm e}^{\rm L}n_{\rm h}. \tag{85}$$

First, we assume an EFM phase. Our strategy in the analysis, is to replace the densities of subpopulations of electrons by their expressions, given in Supplementary Eq. (40). Then instead of $\mu_{\rm e}$, we substitute its expression in terms of $n_{\rm e}$

$$\mu_{\rm e} \approx n_{\rm e}^2/(4D_0^2\Delta_{\rm F}), \tag{86}$$

obtained by inverting Supplementary Eq. (31), where $D_0 = m_*/2\pi$ [see definition below Supplementary Eq. (23)]. We substitute $n_{\rm h} = \kappa/n_{\rm e}$ [cf. Supplementary Eq. (36)] and explicit expressions of $\Lambda_{\rm inter}$ and $\Lambda_{\rm intra}$ [Supplementary Eq. (65) and (68)] in Supplementary Eq. (85) to extract,

$$T_{\rm e} = \left(\frac{6k_{\rm R}^3 v_{\rm s}^3 \kappa n_{\rm e}^2}{4\log^2(2)\Delta_{\rm F}^3 D_0^4}\right)^{1/4}. \tag{87}$$

Note that in Supplementary Eq. (87), $T_{\rm e}$ is expressed as a function of $n_{\rm e}$. Recall, however, that $n_{\rm e}$ is an explicit function of Δn and κ, as follows from Supplementary Eq. (38). Therefore, Supplementary Eq. (87) expresses $T_{\rm e}$ as a function of κ and Δn. The power 1/4 in Supplementary Eq. (87) arises from the LHS term in Supplementary Eq. (85). This term is proportional to $T_{\rm e}^4$, as follows from the definitions of $\Lambda_{\rm intra}$ [see Supplementary Eq. (68)], $n_{\rm e}^{\rm H}$, and $\bar{n}_{\rm e}^{\rm L}$ [see Supplementary Eq. (40)]. Finally, we divide the expression for $\mu_{\rm e}$ given in Supplementary Eq. (86) by $T_{\rm e}$ given in Supplementary Eq. (87) to arrive at

$$\frac{\mu_{\rm e}}{T_{\rm e}} = \left(\frac{C_{\rm EFM}\, n_{\rm e}^6}{\Delta_{\rm F} D_0^4 k_{\rm R}^3 v_{\rm s}^3 \kappa}\right)^{1/4}, \tag{88}$$

where $C_{\rm EFM} = \log^2(2)/384 \approx 0.00125$. In Supplementary Eq. (88), $n_{\rm e}$ can be expressed as a function of Δn and κ, using Supplementary Eq. (38).

Next, we solve Supplementary Eq. (85) in the EFI phase. Here, we replace the electron subpopulation densities by expressions given in Supplementary Eq. (41) and substitute $n_{\rm h} = \kappa/n_{\rm e}$ [cf. Supplementary Eq. (36)]. We then extract an expression for $T_{\rm e}$ from the resulting equation, leading to

$$T_{\rm e} = \left(\frac{3{\rm erf}(1)}{{\rm erfc}(1)} \frac{k_{\rm R}^3 v_{\rm s}^3 \kappa}{\Delta_{\rm F}^{3/2} D_0 n_{\rm e}}\right)^{2/5}. \tag{89}$$

Next, we find $z_{\rm e}$, using

$$z_{\rm e} = n_{\rm e}/(\sqrt{\pi} T_{\rm e} D_{\rm F}(T_{\rm e})) \tag{90}$$

obtained from Supplementary Eq. (32). We substitute Supplementary Eq. (89) in Supplementary Eq. (90) to obtain

$$z_{\rm e} \approx \left(\frac{C_{\rm EFI}\, n_{\rm e}^6}{\Delta_{\rm F} D_0^4 k_{\rm R}^3 v_{\rm s}^3 \kappa}\right)^{1/5}, \tag{91}$$

where $C_{\rm EFI} = {\rm erfc}(1)/(3\pi^{5/2}{\rm erf}(1)) \approx 0.0036$. Recall that $\mu_{\rm e}/T_{\rm e}$ can be extracted from $z_{\rm e}$ using the relation $\mu_{\rm e}/T_{\rm e} = \log(z_{\rm e})$.

We note that the phenomenological analysis outlined in this chapter is aimed to only capture the right scaling of $T_{\rm e}$ and $\mu_{\rm e}$ as functions of the parameters of the system (i.e., κ, Δn, $v_{\rm s}$, etc.), and not the values of the numerical coefficients $C_{\rm EFM}$ and $C_{\rm EFI}$. We use these coefficients as fitting parameters in Fig. 3 in the main text. The best fit is obtained when $C_{\rm EFM} = C_{\rm EFI} \equiv C$.

Next, we consider the distribution of holes in the LFB. The analysis of Supplementary Eq. (45) in the regime $\gamma_{\rm ee} = 0$ is similar to the analysis of Supplementary Eq. (85) in the EFI phase, above. The density of holes and their subpopulations are given by Supplementary Eqs. (33) and (43). In this case, $T_{\rm h}$ and $z_{\rm h}$ are given by

$$T_{\rm h} = \left(\frac{3{\rm erf}(1)}{{\rm erfc}(1)} \frac{k_{\rm R}^3 v_{\rm s}^3 \kappa}{\Delta_{\rm F}^{3/2} D_0 n_{\rm h}}\right)^{2/5}, \; z_{\rm h} \approx \left(\frac{C_{\rm EFI}\, n_{\rm h}^6}{\Delta_{\rm F} D_0^4 k_{\rm R}^3 v_{\rm s}^3 \kappa}\right)^{1/5}. \tag{92}$$

Finally, we verify that Supplementary Eq. (92) is consistent with an assumption of the non-degenerate hole distribution, when the system is doped with electrons. To this end, we show that $z_{\rm h} \to 0$ when we take $\kappa \to 0$ for fixed $\Delta n > 0$. In this limit, $n_{\rm h} \approx \kappa/\Delta n$, see Supplementary Eq. (38). Substituting the latter relation in Supplementary Eq. (92), we find $z_{\rm h} \sim \kappa$. Therefore, the nondegenerate-hole-gas assumption is consistent; indeed $z_{\rm h} \to 0$ as $\kappa \to 0$. Interestingly, note that this assumption is not consistent with Supplementary Eq. (92) when the system is substantially doped with holes, i.e., when $\Delta n \ll -\kappa$. In this case, $n_{\rm h} \approx |\Delta n|$, leading to $z_{\rm h} \sim \kappa^{-1/5}$, i.e., $z_{\rm h} \to \infty$ as $\kappa \to 0$. In fact, when the system is substantially hole doped, the holes form a degenerate distribution analogous to the EFM phase for electrons in an electron-doped system.

2. *Electron and hole temperatures in the presence of electron-electron scattering*

In subsection Supplementary Note 3 h 1 we considered the case $\gamma_{\rm ee} = 0$, for which electron-electron interactions do not equilibrate the electron and hole distributions. Now, we consider the regime where the electron-electron scattering is significant, i.e., when $\gamma_{\rm ee}$ is finite. In this regime, both Supplementary Eqs. (44) and (45) depend on $T_{\rm e}$ and $T_{\rm h}$ and need to be solved jointly. Supplementary Fig. 6 shows $T_{\rm e}$ and $T_{\rm h}$ obtained from a numerical solution of Supplementary Eqs. (44) and (45) in the EFM and EFI regimes. Here, we treat $\gamma_{\rm ee}$ as a constant varied from $\gamma_{\rm ee} = 0$ to large values of $\gamma_{\rm ee}$, where interband thermalization is significant. When $\gamma_{\rm ee} = 0$, the electron and hole temperatures are found in Supplementary Note 3 h 1. Here, we denote by $T_{\rm e,0}$ the temperature of the distribution of the electrons given in Supplementary Eq. (87) if the electrons exhibit an EFM phase and Supplementary Eq. (89) if they exhibit an EFI phase. Likewise, we denote by $T_{\rm h,0}$ the temperature of the distribution of holes for $\gamma_{\rm ee} = 0$, given in Supplementary Eq. (92).

As $\gamma_{\rm ee}$ is increased, the difference between the temperatures of the two distribution is reduced until, in the limit $\gamma_{\rm ee} \to \infty$, they reach a common temperature, T_∞. Predictably, the minority population is more affected by

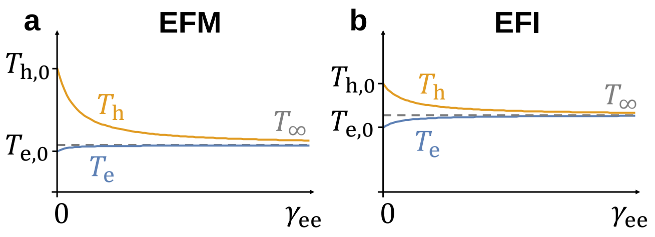

Supplementary Figure 6. **Equilibration of the electron and hole distribution due to electron-electron scattering.** The temperatures of electrons in the UFB (T_e) and holes in the LFB (T_h) are obtained from the solution to Supplementary Eqs. (44) and (45), as a function of γ_{ee} for typical system's parameters in **a** the EFM phase and **b** EFI phase. The temperatures $T_{e,0}$ and $T_{h,0}$ obtained at $\gamma_{ee} = 0$ are given in Supplementary Eqs. (87), (89) and (92). The temperature for $\gamma_{ee} \to \infty$, T_∞, [given in Supplementary Eqs. (95) and (96)] is indicated by the dashed line.

this equilibration, while the temperature of the majority population is only slightly changed. For large values of γ_{ee}, as T_h tends towards T_∞, the difference between $T_h(\gamma_{ee})$ and $T_{h,0}$ is proportional to $\sim 1/\gamma_{ee}$. In what follows, we solve analytically Supplementary Eqs. (44) and (45) in the limit $\gamma_{ee} \to \infty$, to obtain an analytical expression for T_∞ in the EFM and EFI regimes. In this limit, the electron and hole temperatures are almost the same, $|\Delta T| \ll T$ [see discussion above Supplementary Eq. (44)]. Assuming this is the case, we can take only zeroth order in ΔT in all the terms in Supplementary Eqs. (44) and (45), except of the terms proportional to γ_{ee}. Note that the term proportional to γ_{ee} is the same in both of these equations (up to a sign), thus cancels out upon addition of the two equations. The sum of Supplementary Eqs. (44) and (45) then reads,

$$\Lambda_{\rm intra}(T_\infty)(n_e^H \bar{n}_e^L + n_h^H \bar{n}_h^L) = \Lambda_{\rm inter}(n_e^L n_h + n_h^L n_e). \quad (93)$$

Importantly, all the terms in Supplementary Eq. (93) depend only on the mean temperature, T_∞, defined as $T_\infty = \lim_{\gamma_{ee}\to\infty} T$, where in this limit, $\Delta T = 0$. In what follows, we use Supplementary Eq. (93) to determine T_∞. Similarly, ΔT can be found from the difference between Supplementary Eqs. (44) and (45).

First, consider an EFM regime for the electrons. To simplify Supplementary Eq. (93), we neglect the term proportional to $n_h^H \bar{n}_h^L$ relative to $n_e^H \bar{n}_e^L$ in the limit $\kappa \to 0$. We also approximate $n_e^L \approx n_e$ and use $n_h^L = {\rm erf}(1) n_h$ [cf. Supplementary Eqs. (40) and (43)]. As a result, Supplementary Eq. (93) simplifies to

$$\Lambda_{\rm intra}(T_\infty) n_e^H \bar{n}_e^L = C' \Lambda_{\rm inter} n_e n_h, \quad (94)$$

where $C' = 1 + {\rm erf}(1) \approx 1.84$. Note that Supplementary Eq. (94) is reminiscent of Supplementary Eq. (85) up to a constant, C'. Subsequently, the mean temperature obtained from the solution to Supplementary Eq. (94) is related to the solution to Supplementary Eq. (85) by

$$T_\infty = (C')^{1/4} T_{e,0}, \quad (95)$$

where $T_{e,0}$ is given in Supplementary Eq. (87).

Now, we turn to the analysis of the EFI regime. This regime always arises at low-electron doping, which also includes half-filling (where $n_e = n_h$). Therefore in this case, we can not neglect the hole density with respect to the electron one, in Supplementary Eq. (93), as we did in the analysis of the EFM regime, above. To extract T_∞ from Supplementary Eq. (93), we replace the densities of subpopulations by their expressions given in Eqs (41) and (43) in terms of n_e, n_h and T_∞ [substituting T_∞ instead of T_e and T_h]. An extraction of T_∞ from the resulting equation yields

$$T_\infty = \left(\frac{2n_e}{n_e + n_h}\right)^{5/2} T_{e,0}, \quad (96)$$

where $T_{e,0}$ is given in Supplementary Eq. (89). At half-filling $T_\infty = T_{e,0}$, and away from the half-filling, as $n_h/n_e \to 0$, $T_\infty \to 2^{2/5} T_{e,0}$.

To conclude, in this section we showed that for an electron doped system, and in the presence of strong electron-electron scattering, the temperature of the distribution of the electrons in the UFB is close to the temperature obtained by setting $\gamma_{ee} = 0$, given by Supplementary Eqs. (87) and (89). In contrast, the temperature of the distribution of the holes may substantially change due to electron-electron scattering relative to temperature obtained for $\gamma_{ee} = 0$. However the properties of the distribution of holes in the LFB only weakly effect the critical interaction strength, and therefore the phase diagram of the system [cf. Fig. 2a in the main text] (which is mainly set by the properties of the distribution of the electrons in the UFB). Therefore, we expect the phase diagram obtained for $\gamma_{ee} = 0$ to give a good description of the phase diagram of the system in the presence of a nonzero value of γ_{ee}.

i. Interpolation between the EFM and the EFI regimes

In Supplementary Note 3 h 1, we obtained expressions for μ_e/T_e in the EFM and EFI regimes for $\gamma_{ee} = 0$, see Supplementary Eqs. (88) and (91). Here we discuss an interpolation between the two equations which we used to fit the numerical data in Fig. 3 in the main text. We assume $C_{\rm EFM} = C_{\rm EFI} \equiv C$, which yields the best fit for the data in Fig. 3, and define

$$x_e \equiv \zeta n_e^6/(v_s^3 \kappa), \quad (97)$$

where $\zeta = C/(\Delta_F D_0^4 k_R^3)$. Using Supplementary Eq. (97), we rewrite Supplementary Eqs. (88) and (91) as $\mu_e/T_e \approx x_e^{1/4}$ in the EFM regime and $e^{\mu_e/T_e} \approx x_e^{1/5}$ in the EFI regime. The transition between the two regimes occurs in the crossover area, corresponding to $\mu_e/T_e = \mathcal{O}(1)$.

Now we introduce an analytic function which interpolates between the values of μ_e/T_e in the two regimes. This

function needs to interpolate between power-law (in the EFM regime) and exponential (in the EFI regime) functions. Therefore, we expect it to be of the form of the complete Fermi-Dirac integral function,

$$\mathcal{F}_j(\lambda\mu_e/T_e) = x_e^\eta. \tag{98}$$

Here $\mathcal{F}_j(x) = \frac{1}{\Gamma(j+1)}\int_0^\infty \frac{t^j}{e^{t-x}+1}dt$, is the complete Fermi-Dirac integral and $\Gamma(j+1) = j\Gamma(j)$; $\Gamma(1) = 1$ is the Euler gamma function. The function in the LHS of Supplementary Eq. (98) has the following asymptotic values[7],

$$\lim_{\mu_e/T_e\to-\infty} \mathcal{F}_j(\lambda\mu_e/T_e) = e^{\lambda\mu_e/T_e} \tag{99a}$$

$$\lim_{\mu_e/T_e\to\infty} \mathcal{F}_j(\lambda\mu_e/T_e) = \frac{(\lambda\mu_e/T_e)^{j+1}}{\Gamma(j+2)}. \tag{99b}$$

To obtain the right constants in the two asymptotic limits to match the dependencies in the EFM and EFI regimes, we require $\eta/\lambda = 1/5$, $\eta/(j+1) = 1/4$, and $\lambda^{j+1} = \Gamma(j+2)$. These conditions have a unique solution $j \approx -0.304$, $\lambda \approx 0.871$ and $\eta \approx 0.174$.

In order to use the interpolation in Fig. 3c in the main text, we need to obtain an expression for μ_e/T_e as a function of Δn. To this end, we invert Supplementary Eq. (98), and employ the definition of x_e [Supplementary Eq. (97)] to obtain

$$\mu_e/T_e = (1/\lambda)\mathcal{F}_j^{-1}\left\{[\zeta n_e^6/(v_s^3\kappa)]^\eta\right\}, \tag{100}$$

where $\mathcal{F}_j^{-1}[\mathcal{F}_j(x)] = x$. Finally, we replace n_e by a function of Δn and κ, given in Supplementary Eq. (38). We fit the data in Fig. 3c by Supplementary Eq. (100) with a single fitting parameter ζ. The same fitting parameter is used for all the curves shown in Fig. 3 in the main text. In order to fit the data in Figs. 3b and 3d in the main text, we used the equation for $\tilde{U}_c$ [Eq. (10) in the main text], where we substituted Supplementary Eq. (100) for μ_e/T_e appearing inside the $\tilde{\Theta}$-function. Eq. (10) in the main text depends also on μ_h/T_h, which from the analysis similar to the one leading to Supplementary Eq. (100), yields

$$\mu_h/T_h = (1/\lambda)\mathcal{F}_j^{-1}\left\{[\zeta n_h^6/(v_s^3\kappa)]^\eta\right\}. \tag{101}$$

The fit in Figs. 3b and 3d in the main text is performed with the same value of ζ as in Fig. 3c in the main text. We used $\tilde{U}_{ex}$ and $\tilde{U}_{fs}$ as additional fitting parameters.

j. Evaluation of the optimal doping, Δn_*

Finally, we evaluate the optimal doping Δn_*, at which $\tilde{U}_c$ exhibits a deep [cf. Figs. 3b and d in the main text]. The optimal doping is defined by

$$\left.\frac{\partial\tilde{U}_c}{\partial\Delta n}\right|_{\Delta n=\Delta n_*} = 0, \tag{102}$$

where $\tilde{U}_c$ is given in Eq. (10) in the main text, and we estimate μ_e/T_e and μ_h/T_h by Supplementary Eqs. (100) and (101) and n_e and n_h by Supplementary Eq. (38). For simplicity, we neglect the term proportional to $\tilde{U}_{fs}$ as it is constant in Δn, and neglect the term proportional to the density of holes, as its contribution is negligible in the electron-doped system. The approximate expression for $\tilde{U}_c$ then reads

$$\tilde{U}_c \approx \tilde{n}_e/\tilde{\Theta}(\mu_e/T_e). \tag{103}$$

As κ is a weak function of Δn near the phase boundary (see Supplementary Fig. 10), we neglect its derivative with respect to Δn. Therefore, instead of finding the minimum with respect to Δn, we can find the minimum with respect to n_e [which according to Supplementary Eq. (38) is only a function of κ and Δn] for fixed κ. Replacing the Δn-derivative in Supplementary Eq. (102) by n_e-derivative and using the approximate form of $\tilde{U}_c$ given in Supplementary Eq. (103), we arrive at

$$\frac{\tilde{\Theta}'(\mu_e/T_e)}{\tilde{\Theta}(\mu_e/T_e)} = \frac{\lambda}{6\eta}\frac{\mathcal{F}_j'(\lambda\mu_e/T_e)}{\mathcal{F}_j(\lambda\mu_e/T_e)}, \tag{104}$$

where $\tilde{\Theta}'(x) = \frac{d}{dx}\tilde{\Theta}(x)$ and $\mathcal{F}_j'(x) = \frac{d}{dx}\mathcal{F}_j(x)$. Numerical solution of Supplementary Eq. (104) leads to $\mu_e/T_e \approx 1.13$ which, using Supplementary Eq. (98), corresponds to $x_e = c_*$, where $c_* \approx 2.03$. Finally, using Supplementary Eq. (97), we find the density at the optimal doping,

$$n_{e,*} = (c_* v_s^3\kappa/\zeta)^{1/6}. \tag{105}$$

The optimal doping as a function of $n_{e,*}$ is given by $\Delta n_* = n_{e,*} - \kappa/n_{e,*}$ [as follows from Supplementary Eq. (38)]. In the limit $\kappa \to 0$, this simplifies to $\Delta n_* \approx n_{e,*}$.

Supplementary Note 4. PHASE TRANSITION IN THE PRESENCE OF TWO TIME-REVERSAL PARTNERS

In the main text and throughout this supplement, we have discussed a model describing half of the degrees of freedom of a time-reversal symmetric semiconductor [see Eq. (2) in the main text]. In this section, we generalize this model to a model for a material which is time-reversal symmetric (absent the drive). The model includes two copies considered in Eq. (2), which are related by time reversal symmetry. This model is described by the Hamiltonian

$$\hat{\mathcal{H}}^{TR}(t) = \sum_{\boldsymbol{k}} \hat{\mathbf{c}}_{\boldsymbol{k}}^\dagger H_0^{TR}(\boldsymbol{k},t)\hat{\mathbf{c}}_{\boldsymbol{k}} + \hat{\mathcal{H}}_{intra}^{TR} + \hat{\mathcal{H}}_{inter}^{TR}, \tag{106}$$

where $\hat{\mathbf{c}}_{\boldsymbol{k}}^\dagger = (\hat{c}_{\boldsymbol{k}\uparrow1}^\dagger, \hat{c}_{\boldsymbol{k}\downarrow1}^\dagger, \hat{c}_{\boldsymbol{k}\uparrow2}^\dagger, \hat{c}_{\boldsymbol{k}\downarrow2}^\dagger)$, is a four-dimensional spinor; $\sigma = \{\uparrow,\downarrow\}$ denotes the pseudospin degree of freedom and $\tau = \{1,2\}$ are two components related by time

reversal. The non-interacting part of the Hamiltonian reads

$$H_0^{\rm TR}(\boldsymbol{k},t)=\begin{pmatrix}H_0(\boldsymbol{k})+H_{\rm d}(t) & 0\\ 0 & H_0^*(-\boldsymbol{k})+H_{\rm d}(t)\end{pmatrix}, \tag{107}$$

where $H_0(\boldsymbol{k}) = E_0 + (|\boldsymbol{k}|^2/2m_* + E_{\rm g}/2) + \lambda_0\boldsymbol{k}\cdot\boldsymbol{\sigma}$ and $H_{\rm d}(t) = V\cos(\Omega t)\boldsymbol{\sigma}^z$, in accordance with the definitions in Eq. (1) in the main text. We consider contact interaction of electrons of the same time-reversal components,

$$\hat{\mathcal{H}}^{\rm TR}_{\rm intra}=\int d^2\boldsymbol{r}\sum_{\tau=1,2}U\hat{n}_{\uparrow\tau}(\boldsymbol{r})\hat{n}_{\downarrow\tau}(\boldsymbol{r}), \tag{108}$$

and contact interaction between the components

$$\hat{\mathcal{H}}^{\rm TR}_{\rm inter}=\int d^2\boldsymbol{r}U_{12}\hat{n}_1(\boldsymbol{r})\hat{n}_2(\boldsymbol{r}), \tag{109}$$

where $\hat{n}_{\sigma\tau}(\boldsymbol{r}) = \int\frac{d^2\boldsymbol{q}}{(2\pi)^2}e^{i\boldsymbol{q}\cdot\boldsymbol{r}}\hat{c}^\dagger_{\boldsymbol{k}+\boldsymbol{q}\sigma\tau}\hat{c}_{\boldsymbol{k}\sigma\tau}$ and $\hat{n}_\tau(\boldsymbol{r}) = \hat{n}_{\uparrow\tau}(\boldsymbol{r}) + \hat{n}_{\downarrow\tau}(\boldsymbol{r}) - n_0/2$; n_0 is the density of electrons at half-filling.

We will study the system described by Supplementary Eq. (106) using the mean-field approximation. In particular, we consider two independent magnetizations and densities for each of the time-reversal partners ($\alpha = 1,2$),

$$\boldsymbol{h}_\alpha(t)=-\frac{U}{\varpi}\sum_{\boldsymbol{k}}\langle\mathbf{c}^\dagger_{\boldsymbol{k}}(\boldsymbol{\sigma}\otimes\tau_\alpha)\mathbf{c}_{\boldsymbol{k}}\rangle \tag{110a}$$

$$n_\alpha=\frac{1}{\varpi}\int d^2\boldsymbol{r}\langle\hat{n}_\alpha(\boldsymbol{r})\rangle, \tag{110b}$$

where ϖ is the volume of the system and $\tau_{1(2)} = \begin{pmatrix}1(0) & 0\\ 0 & 0(1)\end{pmatrix}$. Such a choice of an order parameter gives rise to the following mean-field Hamiltonian, $\hat{\mathcal{H}}^{\rm TR}_{\rm MF}(t) = \sum_{\boldsymbol{k}}\hat{\mathbf{c}}^\dagger_{\boldsymbol{k}}[H_0^{\rm TR}(\boldsymbol{k},t)+H^{\rm TR}_{\rm mag}(t)]\hat{\mathbf{c}}_{\boldsymbol{k}} + \varpi U_{12}n_1n_2$, where

$$H^{\rm TR}_{\rm mag}(t)=\begin{pmatrix}\boldsymbol{h}_1(t)\cdot\boldsymbol{\sigma} & 0\\ 0 & \boldsymbol{h}_2(t)\cdot\boldsymbol{\sigma}\end{pmatrix}. \tag{111}$$

For simplicity, we will treat the problem as that of an equilibrium system in the rotating frame using the rotating wave approximation (RWA) (see Supplementary Note 1) and minimize quasienergy with the mean-field band structure. This situation corresponds to $\kappa = 0$, i.e., a situation when the Floquet-Umklapp terms are neglected. Our goal is to find $\boldsymbol{h}_1(t)$, $\boldsymbol{h}_2(t)$, n_1 and n_2 such that the ground state of $H^{\rm TR}_{\rm MF}$ will minimize the full interacting Hamiltonian, given in Supplementary Eq. (106). We work in the limit of small doping, $\Delta n \equiv n_1 + n_2 \ll \mathcal{A}_{\rm R}$.

Our choice of the order parameter [Supplementary Eq. (110)] leads to a mean-field Hamiltonian in which the two components related by time reversal are decoupled, for a state with fixed n_1 and n_2. Therefore, each of these components can be analyzed independently. We consider circularly polarized magnetizations for both of them, $\boldsymbol{h}_\tau(t) = h_\tau e^{i\Omega t}(\hat{\boldsymbol{x}} - i\hat{\boldsymbol{y}})/2 + c.c.$ (see discussion following Eq. (8) in the main text). The phase of h_τ spontaneously breaks the rotational symmetry individually exhibited by each partner. In what follows we will present the analysis of the first component ($\tau = 1$); the analysis of its partner ($\tau = 2$) is identical. Our analysis partially follows the analysis presented in Ref. 8. We choose real and positive h_1 leading to the magnetization $h_1\hat{\boldsymbol{x}}$ in the RWA, see Supplementary Eq. (13). The single-particle bandstructure in the mean-field and RWA is given by $\varepsilon_{\boldsymbol{k},\pm} = \pm\varepsilon_{\boldsymbol{k}}$ [see Supplementary Eq. (7)], where

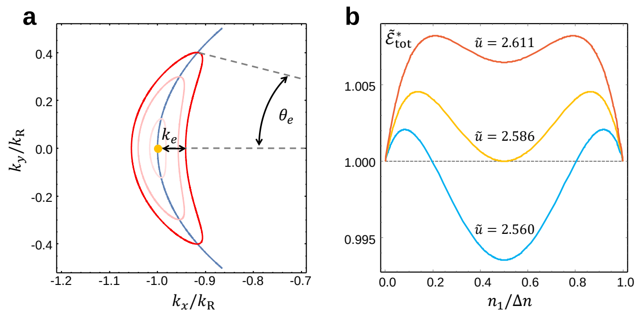

Supplementary Figure 7. **Phase transition in the presence of two time-reversal partners.** **a** Fermi surface of one of the time-reversal partners in the symmetry broken phase, for three densities of electrons. The orange dot indicates the band-minimum point, $\boldsymbol{k}_0$ around which we expand the quasienergy. The opening angle (θ_e) and the width of the oval-shaped Fermi surface (k_e) are indicated on the figure. **b** The normalized optimal quasienergy, $\tilde{\mathcal{E}}^*_{\rm tot}$, given in Supplementary Eq. (119), as a function of the normalized density $n_1/\Delta n$, for three values of the normalized inter-component interaction $\tilde{u}$. For $\tilde{u} < 4-\sqrt{2} \approx 2.586$ the total energy has a single global minimum point at $n_1 = \Delta n/2$, corresponding to equal density of particles in the two time-reversal partners. For $\tilde{u} > 4-\sqrt{2}$, the total energy has two global minimum points at $n_1 = 0$ and $n_1 = \Delta n$. In this case, one of the partners is fully occupied and the other is empty due to strong inter-partner repulsion. The two cases are separated by a critical point at $\tilde{u} = 4-\sqrt{2}$, where the three possibilities coexist.

$$\varepsilon_{\boldsymbol{k}}=\sqrt{\left(\frac{k^2}{2m_*}-\frac{\delta E}{2}\right)^2+\left(\frac{\Delta_{\rm F}k_x}{2k_R}+h_1\right)^2+\left(\frac{\Delta_{\rm F}k_y}{2k_R}\right)^2}. \tag{112}$$

The Fermi surface, corresponding to a fixed density n_1, has a curved elliptic shape centered around the band-minimum point, $\boldsymbol{k}_0 \equiv -k_{\rm R}\hat{\boldsymbol{x}}$, see Supplementary Fig. 7a. We expand $\varepsilon_{\boldsymbol{k}}$ around $\boldsymbol{k}_0$ in polar coordinates, $k_x = k\cos(\theta)$, $k_y = k\sin(\theta)$, for small θ and $\delta k = k - k_R$. For simplicity, we assume $\Delta_{\rm F} \ll \delta E$ and $h_1 \ll k_R^2/2m_*$ leading, up to the second order in δk and θ, to

$$\varepsilon_{\boldsymbol{k}}\approx\Delta_{\rm F}/2-h_1+\frac{\delta E}{\Delta_{\rm F}}\frac{\delta k^2}{m_*}+\frac{h_1}{2}\theta^2. \tag{113}$$

We define the kinetic energy density by $\mathcal{E}_{\rm kin} = \int\frac{d^2\boldsymbol{k}}{(2p)^2}(\varepsilon_{\boldsymbol{k}} - h_1\langle\sigma^x\rangle_{\boldsymbol{k}})$, where $\langle\sigma^x\rangle_{\boldsymbol{k}} = (\Delta_{\rm F}k_x/2k_{\rm R} +$

$h_1)/\varepsilon_{\boldsymbol{k}}$. In the definition of $\mathcal{E}_{\rm kin}$ we subtracted the term proportional to h_1 to avoid double counting of the magnetization energy, as it will be accounted in the interaction energy density $\mathcal{E}_{\rm int}$, see below. The expansion of $\langle\sigma^x\rangle_{\boldsymbol{k}}$ up to the second order in δk and θ reads

$$\langle\sigma^x\rangle_{\boldsymbol{k}} \approx -1 + \frac{\Delta_{\rm F}\delta k}{2k_{\rm R}(\Delta_{\rm F}/2 - h_1)} + \\ + \frac{\delta E \delta k^2}{m_* \Delta_{\rm F}(\Delta_{\rm F}/2 - h_1)} + \left(\frac{1}{2} + \frac{h_1}{\Delta_{\rm F}/2 - h_1}\right)\theta^2. \tag{114}$$

The integral of $\mathcal{E}_{\rm kin}$ is over a curved elliptical area in k-space enclosed by the Fermi surface, characterized by the radial length k_e and the angular aperture θ_e, see Supplementary Fig. 7a. We parametrize this area by $\delta k = k_e x \cos(y)$ and $\theta = \theta_e x \sin(y)$, where $x \in [0,1]$ and $y \in [0, 2\pi]$. By geometrical constraints, the values of k_e and θ_e are fixed by the density and the magnetization magnitude as follows,

$$k_e\theta_e = \frac{4\pi n_1}{k_{\rm R}} \tag{115a}$$

$$\frac{k_e^2}{\theta_e^2} = \frac{\Delta_{\rm F}}{\delta E}\frac{m_* h_1}{2}. \tag{115b}$$

Using the new variables, the kinetic energy density reads $\mathcal{E}_{\rm kin} = \frac{k_e k_{\rm R}\theta_e}{(2\pi)^2}\int_0^1 x dx \int_0^{2\pi} dy(\varepsilon_{\boldsymbol{k}} - h_1\langle\sigma^x\rangle_{\boldsymbol{k}})$. Integration over x and y [the dependence of $\varepsilon_{\boldsymbol{k}}$ and $\langle\sigma^x\rangle_{\boldsymbol{k}}$ on x and y is given by Supplementary Eqs. (113) and (114)] yields

$$\mathcal{E}_{\rm kin}(n_1, h_1) \approx \frac{\Delta_{\rm F} n_1}{2} + \sqrt{\frac{h_1}{2\Delta_{\rm F}}}\frac{\pi n_1^2}{m_*} + \mathcal{O}(n_1^2 h_1^{3/2}). \tag{116}$$

Next, we evaluate the interaction energy density due to interaction of electron-electron interaction given in Supplementary Eq. (108), $\mathcal{E}_{\rm int} = \frac{U}{\varpi^2}\sum_{\boldsymbol{qkk'}}\langle \hat{c}^\dagger_{\boldsymbol{k+q}\uparrow}\hat{c}_{\boldsymbol{k}\uparrow}\hat{c}^\dagger_{\boldsymbol{k'-q}\downarrow}\hat{c}_{\boldsymbol{k'}\downarrow}\rangle$. Using the Wick's theorem the interaction energy density simplifies to $\mathcal{E}_{\rm int} = \frac{U}{4}(n_1^2 - \langle\sigma^x\rangle^2 - \langle\sigma^y\rangle^2 - \langle\sigma^z\rangle^2)$, where $\langle\sigma^\alpha\rangle \equiv \int \frac{d^2\boldsymbol{k}}{(2\pi)^2}\langle\sigma^\alpha\rangle_{\boldsymbol{k}}$. We further neglect $\langle\sigma^y\rangle^2$ and $\langle\sigma^z\rangle^2$ as they depend on higher powers of h_1 and n_1. Substituting Supplementary Eq. (114) in the definition for $\langle\sigma^x\rangle$, we find $\langle\sigma^x\rangle \approx -n_1 + \frac{\pi n_1^2}{m_*\sqrt{2h_1\Delta_{\rm F}}} + \mathcal{O}(n_1^2 h_1^{1/2})$. Therefore, the interaction energy density reads

$$\mathcal{E}_{\rm int}(n_1, h_1) \approx \frac{U\pi n_1^3}{m_*\sqrt{8h_1\Delta_{\rm F}}} + \mathcal{O}(n_1^4 h_1^{-1}). \tag{117}$$

The kinetic and interaction energy densities of the time-reversal partner obtain expressions similar to Supplementary Eqs. (116) and (117) with n_2 and h_2 replacing n_1 and h_1. We define the full energy density of each of the components ($\tau = 1, 2$) as the sum of the kinetic and the interaction energy densities, $\mathcal{E}_{\rm full}(n_\tau, h_\tau) = \mathcal{E}_{\rm kin}(n_\tau, h_\tau) + \mathcal{E}_{\rm int}(n_\tau, h_\tau)$. Finally, the total energy density includes the full energy densities of both components and the interaction between them [Supplementary Eq. (106)]. The total energy density of the system then reads

$$\mathcal{E}_{\rm tot}(n_1, n_2, h_1, h_2) = \\ = \mathcal{E}_{\rm full}(n_1, h_1) + \mathcal{E}_{\rm full}(n_2, h_2) + U_{12}\, n_1 n_2. \tag{118}$$

Next, we minimize $\mathcal{E}_{\rm tot}$ with respect to h_1, h_2 and n_1 (given $n_2 = \Delta n - n_1$). Due to the separable form of $\mathcal{E}_{\rm tot}$, its minimum with respect to h_1 and h_2 for fixed n_1 and n_2, coincides with the minimum of its respective terms. The minimum of $\mathcal{E}_{\rm full}(n_1, h_1)$ with respect to h_1 yields $h_1^{\rm opt} = \frac{1}{2}Un_1$. Similarly, the minimum of $\mathcal{E}_{\rm full}(n_2, h_2)$ with respect to h_2 yields $h_2^{\rm opt} = \frac{1}{2}Un_2$. Note that $\mathcal{E}_{\rm tot}$ denotes the total energy density relative to the full lower band. In fact, the energy of the full lower band also depends on h_1 and h_2. However, this dependence is weak, hence we neglected the contribution of the full lower band in our calculation of $h_1^{\rm opt}$ and $h_2^{\rm opt}$.

Finally, we define the optimal energy density with kinetic energy measured relative to the band bottom as follows, $\mathcal{E}^*_{\rm tot}(n_1) \equiv \mathcal{E}_{\rm tot}(n_1, \Delta n - n_1, h_1^{\rm opt}, h_2^{\rm opt})$. The minimum of $\mathcal{E}^*_{\rm tot}$ with respect to n_1 yields the filling of each of the components, $n_1^{\rm opt}$ and $n_2^{\rm opt} = \Delta n - n_1^{\rm opt}$ in the variational ground state of the system in the rotating frame. Substituting explicit expressions of the energy densities [Supplementary Eqs. (116) and (117)] and optimal magnetizations ($h_1^{\rm opt}$ and $h_2^{\rm opt}$) into Supplementary Eq. (118), we find $\mathcal{E}^*_{\rm tot}(n_1) = \frac{\pi(\Delta n)^2}{m_*}\sqrt{\frac{U\Delta n}{\Delta_{\rm F}}}\tilde{\mathcal{E}}^*_{\rm tot}(n_1/\Delta n) + \Delta n \Delta_{\rm F}/2$, where

$$\tilde{\mathcal{E}}^*_{\rm tot}(\tilde{n}) = \tilde{n}^{5/2} + (1-\tilde{n})^{5/2} + \tilde{u}\,\tilde{n}(1-\tilde{n}), \tag{119}$$

and $\tilde{u} \equiv \frac{m_* U_{12}}{\pi}\sqrt{\frac{\Delta_{\rm F}}{U\Delta n}}$.

Supplementary Fig. 7b shows $\mathcal{E}^*_{\rm tot}$ as a function of the normalized density $n_1/\Delta n$ for three values of $\tilde{u}$. For $\tilde{u} < 4 - \sqrt{2}$, the quasienergy has a global minimum at $n_1 = \Delta n/2$, corresponding to an equal share of particles between the two time-reversal components, see Supplementary Fig. 7b. For $\tilde{u} > 4 - \sqrt{2}$, the quasienergy density has two global minima at $n_1 = 0$ and $n_1 = \Delta n$, corresponding to the situation where one of the components is undoped due to the strong inter-partner repulsion.

In conclusion, we showed that a model which includes the time reversal partner of the Hamiltonian discussed in the main text [see Eq (1)], may still exhibit a symmetry breaking phase. Within our mean-field analysis above, we found that when the repulsion between the time-reversal partners is not too strong, the symmetry breaking in both time-reversal partners minimizes the energy in the rotating frame. If the inter-partner repulsion is stronger than a critical value, only one of the partners becomes doped and develops a symmetry breaking term. Interestingly, within the mean-field approximation the energy is independent of the relative orientation of the magnetizations of the two time-reversal partners. To determine the optimal relative orientation, one needs to

$A/E_{\rm g}$	1/60	$V/E_{\rm g}$	$\sqrt{0.025}$
$B/E_{\rm g}$	1/30	$\delta E/E_{\rm g}$	1/200
$A'/E_{\rm g}$	1/240	$v_\ell/(\Delta_{\rm F}/k_{\rm R})$	1.6
$B'/E_{\rm g}$	1/120		

Supplementary Table 1. Parameters used in the numerical simulations.

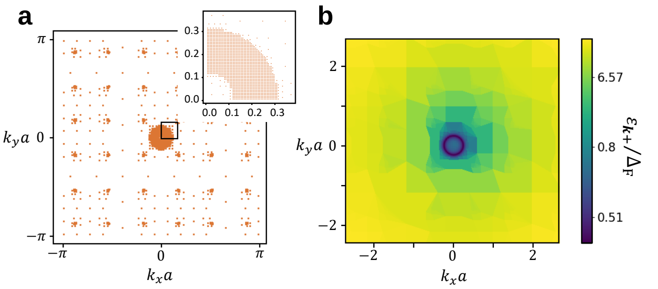

Supplementary Figure 8. **The nonuniform momentum grid.** **a** Illustration of the nonuniform momentum grid used in the simulation. Each dot indicates a point in the momentum grid. The highest density of momentum points resides around the resonance curve, $|\boldsymbol{k}| = k_R$. The total number of points in our simulations is 8008. The inset shows a zoom in on the area with high density of points. **b** The energy of the upper band on the logarithmic scale computed on the nonuniform grid in panel **a**.

go beyond the mean-field theory we considered. We believe this is an interesting problem for future research. In addition, the analysis presented in this section is limited to $\kappa = 0$ case. It would be interesting to extend this analysis to finite effective temperature steady states.

Supplementary Note 5. DETAILS OF THE NUMERICAL SIMULATION

In this section we discuss the details of the numerical analysis. The parameters used in the simulation are summarized in Supplementary Table 1. To see a pronounced suppression in $U_{\rm c}$ (see Fig. 2a in the main text) we worked at low densities of excitations, requiring a high-resolution grid of k-points. To reduce the amount of computational power, we created a nonuniform grid of momentum points with an enhanced density of points around the resonance ring, and total 8008 k-points. Supplementary Fig. 8 demonstrates the grid that we used and the Floquet quasienergy levels of the upper Floquet band near the resonance ring. Each point in panel (a) of this figure indicates a center of a grid cell, of area depending on the density of the grid in the vicinity of this cell. To ensure that the areas of all the cells amount to the total area of the Brillouin zone $[(2\pi/a)^2]$, we built the nonuniform grid as follows. First, we partitioned the Brillouin zone to $2^{10} \times 2^{10}$ cells. Then, we recursively combined clusters of $(2\times 2)^n$ cells to form super cells,

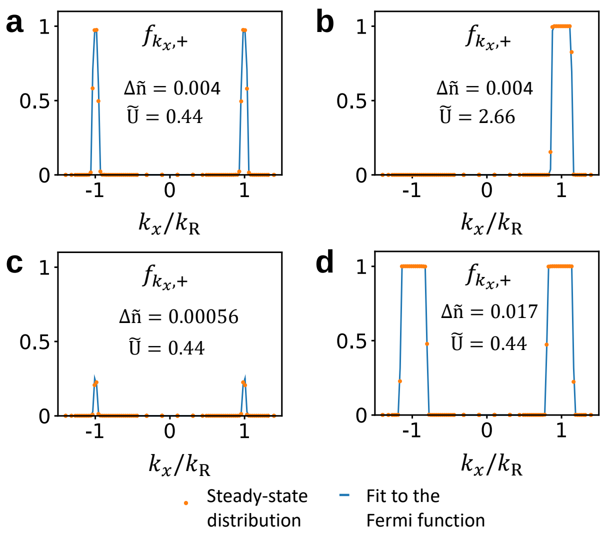

Supplementary Figure 9. **Fit of the steady state to the Fermi Dirac distribution.** Comparison between the steady state distribution of electrons in the upper Floquet band extracted from the numerical simulation, and the fit to the Fermi function, along the cut $k_y = 0$. The values of $\Delta\tilde{n}$ and $\tilde{U}$ are indicated on the panels. **a**, **b** Steady-state distributions at the points indicated by the green and red squares in Fig. 2a in the main text. **c** Low-doping non-degenerate distribution of electrons in the "paramagnetic" phase, corresponding to the EFI regime. **d** Strongly doped "paramagnetic" phase, describing a degenerate Fermi distribution in the EFM regime.

where the value of $n < 10$ controls the density of cells in the vicinity of the cluster. The area of such a cluster equals $(2\pi/(2^{10-n}a))^2$.

The k-grid is used to define the populations and the rates in the kinetic equation [Eq. (4) in the main text]. We calculated the rates $I^{\rm s}_{\boldsymbol{k}\nu}$, $I^{\ell}_{\boldsymbol{k}\nu}$, appearing in this equation, using the Fermi's golden rule,

$$I^p_{\boldsymbol{k}\nu} = \sum_{\boldsymbol{k}'\nu'} (\dot{f}_{\boldsymbol{k}\nu})_{p,\boldsymbol{k}'\nu'}, \qquad (120)$$

where $(\dot{f}_{\boldsymbol{k}\nu})_{p,\boldsymbol{k}'\nu'}$ is given by Supplementary Eq. (46). In a realistic realization of the driven system in a solid state setup, we expect the electron-phonon excitation rates to be suppressed by $(V/\Omega)^2$, as explained in Supplementary Note 3 d 2. However, in our simulation, we took a large value of V/Ω (see Tab. 1) which facilitates large density of states near the Floquet gap. To capture the suppression of the electron-phonon excitations, we multiplied the squared matrix element $\mathcal{P}^{(l)}_{\rm s}$ appearing in the definition of $(\dot{f}_{\boldsymbol{k}\nu})_{{\rm s},\boldsymbol{k}'\nu'}$ [see Supplementary Eq. (46)], by a factor v_V^{2l}, where $v_V = 10^{-2}$ represents realistic values of V/Ω.

To avoid using four-point terms for the electron-electron scattering collision integral in the numerical simulation of the kinetic equation [Supplementary Eq. (50)], we used an approximate expression for the collision integral of the form $I^{\rm ee}_{\boldsymbol{k}\nu} = r_{\rm A} U^2 I^{\ell}_{\boldsymbol{k}\nu}$. Such a collision

integral mimics a source term due to Electron-electron interaction-activated excitation rate, described in Supplementary Note 3 d 3. The parameter r_A sets the relative strength of this rate. Note that our numerical simulation, with the above simplification for the collision integral, does not take in account equilibration of the electron and hole distributions via electron-electron scattering. We argue in Supplementary Note 3 h 2 that including this process will not qualitatively change the results of the numerical simulation.

In the first iteration of the algorithm we set the initial value of the magnetization field to have a form of the in-plane rotating field $\boldsymbol{h}^{(0)}(t) = h_1^{(0)}(\hat{\boldsymbol{x}} - i\hat{\boldsymbol{y}})e^{i\Omega t} + c.c.$, with $|h_1^{(0)}|/\Delta_F \sim 5\times 10^{-7}$. We then solved the kinetic equation [given in Eq. (4) in the main text] in the steady-state ($\dot{f}_{\boldsymbol{k}\nu} = 0$), using the Newton-Raphson method and obtained the distribution $f_{\boldsymbol{k}\nu}$ after the first iteration. We used $f_{\boldsymbol{k}\nu}$ to calculate the magnetization vector $\boldsymbol{h}^{(1)}(t)$ for the next iteration of the algorithm, using Eq. (2) in the main text. As the momentum integral appearing in this equation is very sensitive to finite size effects, we evaluated the magnetization using an adaptive integration method. To this end, we used a fit of the steady-state distribution by two Fermi functions for the electron and hole excitations. Supplementary Fig. 9 shows the comparison of the fits to the numerically obtained distributions at several points in the phase diagram in Fig. 2a in the main text. We then numerically integrate Eq. (2) in the main text over the fitting functions, to obtain the magnetization, $\boldsymbol{h}^{(1)}(t)$. Finally, we substituted $\boldsymbol{h}^{(1)}(t)$ in the mean-field Hamiltonian [Eq. (3) in the main text] and used it for the next iteration of the algorithm. The iteration loop terminates when the change in amplitude of the left-handed circular component of the magnetization reaches a threshold value. We verified that other components have also converged.

a. Relation between κ and system parameters

In the simulation we controlled the value of κ by the variation of the electron-photon coupling coefficient g_ℓ and measured κ from measurements of excitation densities [via Supplementary Eq. (36)]. The definition of κ, given in Supplementary Eq. (37), suggests that it is also sensitive to other parameters of the system controlling the heating and cooling rates. In particular, even though we fixed the value of g_ℓ throughout the phase diagram in Fig. 2a in the main text, the value of κ still may vary as a function of U and Δn. We chose the point $U = 0$, $\Delta n = 0$ in the phase diagram as a reference and denoted the value of κ at this point by κ_0.

The value of κ is approximately equal to κ_0 throughout most of the phase diagram in Fig. 2a in the main text (for fixed g_ℓ). Yet, it differs from κ_0 deep in the broken symmetry phase, as the single-particle Floquet band structure in this regime is significantly deformed. Furthermore, the value of κ slightly varies (at low doping) even in the symmetric phase, due to higher order doping-sensitive terms omitted from Supplementary Eq. (35). Likewise, we expect slight changes in κ relative to κ_0 at the phase boundary. As we show in Supplementary Fig. 10, the relative change in κ at the phase boundary near the optimal doping (indicated by $\Delta\tilde{n}_*$ in the figure) is less than 5%. This finding justifies the assumptions made in our extended rate equation (outlined in Supplementary Note 3), where we treated κ as a doping-independent quantity.

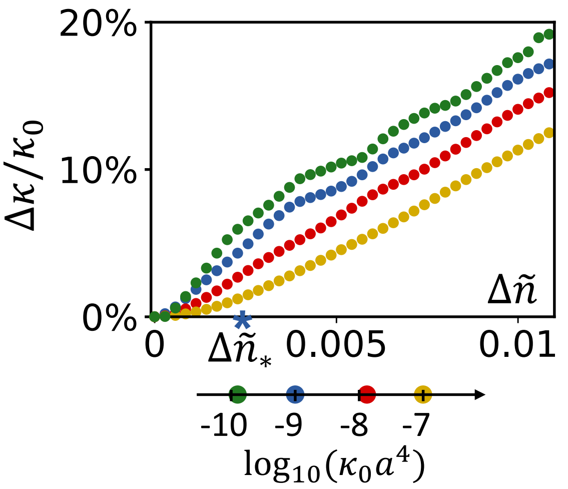

Supplementary Figure 10. **Dependence of κ on the doping.** The difference $\Delta\kappa = \kappa - \kappa_0$ as a function of the doping along the phase boundary line for four values of κ_0 indicated at the logarithmic scale. The optimal doping for $\kappa_0 a^4 \approx 10^{-9}$ is indicated by $\Delta\tilde{n}_*$.

b. Sensitivity of the self-consistent solution to the initial conditions

Here we examine the sensitivity of the self-consistent solution to the initial magnetization, $\boldsymbol{h}^{(0)}(t)$. All the results are obtained for $\Delta\tilde{n} = 0.004$ and $\tilde{U} = 2.66$, indicated by the red square in Fig.2a in the main text.

First, we tested the response to different phase shifts of the initial magnetization, $\boldsymbol{h}^{(0)}(t) = h_1^{(0)} e^{i\varphi_0}(\hat{\boldsymbol{x}} - i\hat{\boldsymbol{y}})e^{i\Omega t} + c.c.$. We swept φ_0 in the range $[-180°, 0°]$, and measured the in-plane component of the first harmonics of the self-consistent mean-field solution, $\boldsymbol{h}_1^{(xy)} = |h_x|e^{i\varphi_x}\hat{\boldsymbol{x}} + |h_y|e^{i\varphi_y}\hat{\boldsymbol{y}}$ [see definition of $\boldsymbol{h}_1^{(xy)}$ in the main text following Eq. (8)]. Our simulation provides the complex-valued amplitudes of the components in the coordinate system spanned by $\hat{\boldsymbol{x}}$ and $\hat{\boldsymbol{y}}$. We define a new coordinate system (spanned by $\hat{\boldsymbol{x}}'$ and $\hat{\boldsymbol{y}}'$) in which the $\hat{\boldsymbol{x}}'$ component

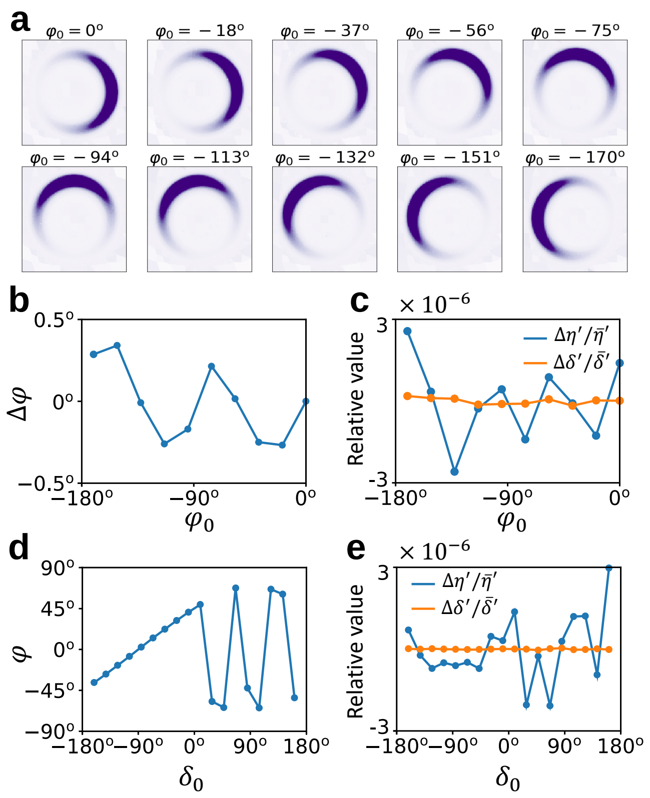

Supplementary Figure 11. **Sensitivity of the self-consistent solution to initial conditions.** All the results are obtained for $\Delta\tilde{n} = 0.004$ and $\tilde{U} = 2.68$, indicated by the red square in Fig. 2a in the main text. **a** Steady-state distribution of the electrons in the upper Floquet band, $f_{\boldsymbol{k}+}$, as a function of k_x and k_y, near the resonance ring for several values of the phase shift in the initial magnetization, φ_0, see text. **b** The angle mismatch between φ_0 and the phase of the self-consistent field, φ, for the data shown in panel **a**. **c** Fluctuations of the relative polarization amplitudes and angles, $\Delta\eta' = \eta' - \bar{\eta}'$, $\Delta\delta' = \delta' - \bar{\delta}'$, where $\bar{\eta}' \approx 1.078$ and $\bar{\delta}' = -90°$. **d** Phase shift of the self-consistent field φ, as a function of the relative initial polarization angle δ_0. **e** Fluctuations of the relative polarization amplitudes and angles as a function of δ_0.

is real. The angle between $\hat{\boldsymbol{x}}$ and $\hat{\boldsymbol{x}}'$ is given by

$$\tan(\varphi) = -\frac{|h_x|\sin(\varphi_x)}{|h_y|\sin(\varphi_y)}. \tag{121}$$

In the new coordinate system, the in-plane component of the magnetization reads

$$\boldsymbol{h}_1^{(xy)} = |h_{x'}|\hat{\boldsymbol{x}}' + |h_{y'}|e^{i\delta'}\hat{\boldsymbol{y}}'. \tag{122}$$

In a system described by a perfectly rotational symmetric interacting Hamiltonian, we expect $\varphi = \varphi_0$. In turn, we expect the $\hat{\boldsymbol{x}}'$ and $\hat{\boldsymbol{y}}'$ components to be same as the magnetization obtained in the case of $\varphi_0 = 0$, shown in Fig. 2b in the main text. Namely, the relative angle between $\hat{\boldsymbol{x}}'$ and $\hat{\boldsymbol{y}}'$ polarizations, δ', is expected to be equal $\sim -90°$. And the ratio of the amplitudes,

$$\eta' = h_{x'}/h_{y'} \tag{123}$$

is expected to be equal ~ 1.078.

Supplementary Fig. 11a demonstrates the steady-state distributions of the electrons as a function of the phase shift, φ_0. Clearly, the angle along which the system breaks the symmetry in the rotating frame, φ, follows φ_0. Supplementary Fig. 11b shows the deviation between the two angles, $\Delta\varphi = \varphi - \varphi_0$ as a function of φ_0. The deviation exhibits fluctuations around 0° within the range of ±0.5° presumably due to the lattice effects, spoiling the perfect rotational symmetry of the Hamiltonian. Supplementary Fig. 11c shows the relative fluctuations of the angle δ' [see Supplementary Eq. (122)], and the ratio of the amplitudes, η' [Supplementary Eq. (123)] as a function of φ_0. Both quantities are almost constant with relative fluctuations of the order of $\sim 10^{-6}$ around their averages over the data set, $\bar{\delta}' \approx -90°$ and $\bar{\eta}' \approx 1.078$.

Next, we checked the stability of the self-consistent solution to different elliptical polarizations of the initial conditions. This time, we set the initial mean-field to $\boldsymbol{h}^{(0)}(t) = h_1^{(0)}(\hat{\boldsymbol{x}} + e^{i\delta_0}\hat{\boldsymbol{y}})e^{i\Omega t} + c.c.$, and swept δ_0 in the range $[-180°, 180°]$. For each value of δ_0, we found the self-consistent mean-field solution and extracted the first harmonic of an in-plane component of the magnetization. The result rotated to the new coordinate system by φ [Supplementary Eq. (121)] is of the form given in Supplementary Eq. (122). Supplementary Fig. 11e shows the fluctuations of δ' and η' [defined in Supplementary Eq. (123)], relative to the averages $\bar{\delta}' \approx -90°$ and $\bar{\eta}' \approx 1.078$, as a function of δ_0. It follows that the quantities δ' and η' are almost insensitive to δ_0, with relative fluctuations of the order of $\sim 10^{-6}$. Supplementary Fig. 11d shows the rotation angle, φ, as a function of δ_0. When $\delta_0 < 0$, the phase shift of the self-consistent solution approximately equals the average between the phases of the initial x and y polarizations, $\varphi \approx (\delta_0 + 90°)/2$. This relation breaks when $\delta_0 > 0$, i.e., when the polarization has a strong right-handed circular polarization component. Exactly at $\delta_0 = 180°$, the self-consistent algorithm does not find a solution with a nonzero magnetization.

Supplementary Note 6. ANALYSIS WHEN THE FLOQUET-AUGER RATE IS SIGNIFICANT

In this section we analyze the phase diagram when the excitation rate due to Floquet-Auger heating described in Supplementary Note 3 d 3 is significant. The effect of Floquet-Auger processes is introduced in the model by an additional heating channel with a strength depending on U^2 (see Supplementary Note 5). In this case, κ reads

$$\kappa(\tilde{U}) = \kappa_{\rm ph} + \chi_{\rm ee}\tilde{U}^2, \tag{124}$$

a	5 Å	$E_{\rm g}$	1 eV
λ_0	15 eVÅ	m_*	0.5 m_0
Ξ	13.7 eV	ρ_m	5300 kg/m^3
$v_{\rm s}$	2000 m/s	τ_ℓ	1 ns
Ω	1.015 eV	$\Delta_{\rm F}$	10 meV

Supplementary Table 2. Parameters used for a typical experimental setup.

where $\chi_{\rm ee} = \kappa_{\rm ee}/\tilde{U}^2$ and $\kappa_{\rm ee} = \Gamma_{\rm ee}/\Lambda_{\rm inter}$ [cf. Supplementary Eqs. (62) and (65)]. Note that $\tilde{U} = \mathcal{A}_{\rm R}U/\delta E$ is the normalized interaction strength, such that $\chi_{\rm ee}$ and $\kappa_{\rm ph}$ have the same dimensions. To see how the phase boundary is affected by the Floquet-Auger processes, recall that $\tilde{U}_{\rm c}$, given by Eq. (10) in the main text, depends on κ through the dependence of the densities $n_{\rm e}$ and $n_{\rm h}$ on κ [see Supplementary Eq. (38)] and through the dependence of $\mu_{\rm e}/T_{\rm e}$ and $\mu_{\rm h}/T_{\rm h}$ on κ, cf. Supplementary Eq. (100). Therefore, Eq. (10) in the main text becomes a transcendental equation for $\tilde{U}_{\rm c}$ which can be solved numerically. The primary effect of a strong Floquet-Auger rate would be increasing the excitation density and heating the steady-state distribution.

We evaluate the relative effect of the Floquet-Auger heating by a dimensionless parameter $R_{\rm A} = \chi_{\rm ee}/\kappa_{\rm ph}$. Supplementary Figs. 12a and b show $\tilde{U}_{\rm c}$ and $\mu_{\rm e}/T_{\rm e}$ resulting from the numerical solution of the transcendental equation obtained from combining Eq. (10) in the main text with Supplementary Eqs. (38), (100), (101) and (124). We plot the results for four values of $R_{\rm A}$. The effect of increasing $R_{\rm A}$ is very similar to the effect of changing κ_0 in Figs. 3c and d in the main text, as both control the effective heating. Panels c and d in Supplementary Fig. 12 show the results of the self-consistent mean-field calculation of the in-plane magnetization component, $\boldsymbol{h}_1^{(xy)}$, as a function of $\tilde{U}$ and $\Delta\tilde{n}$ for $R_{\rm A} = 1$ and $R_{\rm A} = 100$. The parameter $R_{\rm A}$ is controlled in the simulation by tuning $r_{\rm A}$ (see Supplementary Note 5). White dashed lines represent the analytical solution to Eq. (10) in the main text similar to the corresponding line in the panel a.

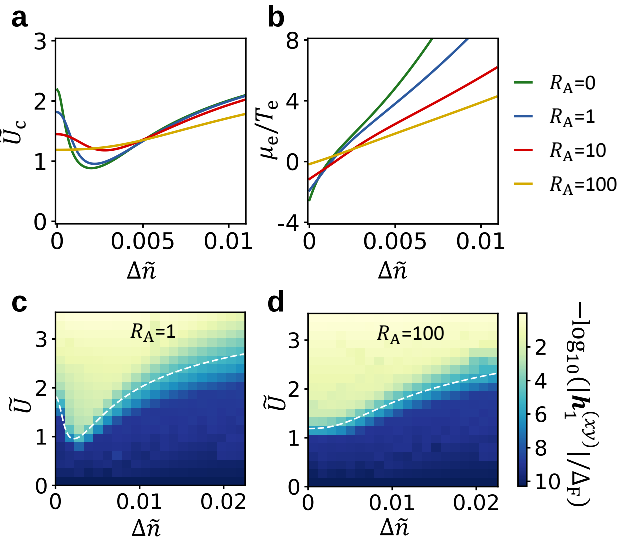

Supplementary Figure 12. **Phase diagram as a function of the strength of the Auger processes.** We define $R_{\rm A} = \chi_{\rm ee}/\kappa_{\rm ph}$ and set $\kappa_{\rm ph} \approx 10^{-9}$ and $v_{\rm s} = 0.0086\Delta_{\rm F}/k_{\rm R}$. **a** $\tilde{U}_{\rm c}$ obtained by solving Eq. (10) in the main text where $n_{\rm e}$, $n_{\rm h}$, $\mu_{\rm e}/T_{\rm e}$, and $\mu_{\rm h}/T_{\rm h}$ were taken from the analytical rate equation approach [Supplementary Eqs. (38), (100) and (101)] and κ is given by Supplementary Eq. (124). We used the same parameters for ζ, $\tilde{U}_{\rm ex}$, and $\tilde{U}_{\rm fb}$ as in Fig. 3 in the main text. **b** The ratio $\mu_{\rm e}/T_{\rm e}$ extracted from Supplementary Eqs. (100) and (124), for the values of $\tilde{U}$ obtained from the data in panel **a**. **c**, **d** Spontaneous magnetization strength, $|\boldsymbol{h}_1^{(xy)}|$ obtained from the self-consistent mean-field calculation, as a function of a normalized electron doping and normalized interaction strength, for two values of $R_{\rm A}$. White dashed lines represent the analytical calculation, shown in panel **a** for the corresponding values of $R_{\rm A}$.

Supplementary Note 7. ESTIMATES FOR A TYPICAL EXPERIMENTAL SETUP

Here we present a detailed calculations of the estimates that we performed in the discussion section in the main text. The parameters for a typical experimental setup are given in Supplementary Table 2. In our estimates we work in the limit $V/\Omega \ll 1$, where $V \approx \Delta_{\rm F}\Omega/(2\lambda_0 k_{\rm R})$. In this parameter regime, radiative recombination is the dominant source of heating: $\Gamma_\ell \gg \Gamma_{\rm s}, \Gamma_{\rm ee}$ [cf. Supplementary Eqs. (56),(60),(62)].

First, we estimate the value of $\kappa \approx \Gamma_\ell/\Lambda_{\rm inter}$. From Supplementary Eq. (65) we have $\Lambda_{\rm inter} \approx (\Delta_{\rm F}\tau_\Lambda^2\mathcal{A}_{\rm BZ})^{-1}$, where $\tau_\Lambda^{-2} = 12\pi^3 g_{\rm s}^2\rho_{\rm s}^0 k_{\rm R}^2$ and $\mathcal{A}_{\rm BZ} = (2\pi/a)^2$. The electron-phonon coupling coefficient and the phonon density of states are given by $g_{\rm s}^2 = \Xi^2/(2\rho_m a^2)$ (see, e.g., Ref. 9) and $\rho_{\rm s}^0 = 1/(\pi v_{\rm s})$, respectively. Furthermore, the resonance momentum $k_{\rm R}$ is approximately $(\delta E m_*)^{\frac{1}{2}}$, where $\delta E = \Omega - E_{\rm g}$. We also approximate $\Gamma_\ell \approx \mathcal{A}_{\rm R}/\tau_\ell$, where $\mathcal{A}_{\rm R} = \pi k_{\rm R}^2$, see Supplementary Note 3 d. Combining the estimates for Γ_ℓ and $\Lambda_{\rm inter}$, we arrive at $\kappa \approx 3 \times 10^{-7}\mathcal{A}_{\rm BZ}^2$.

We use the value of Γ_ℓ above to estimate the energy flux dissipated by the heat baths. The energy flux density dissipated by the phonon and photon heat baths is given respectively by $W_{\rm s} = \Gamma_\ell\delta E$ and $W_\ell = \Gamma_\ell E_{\rm g}$. For a system of area L^2, where $L = 5\ \mu m$, we obtain $W_{\rm s}L^2 \approx 60\ \mu W$ and $W_\ell \approx 4\ mW$. To estimate the operation temperature of the cryogenic refrigerator, we employ Supplementary Eq. (87). For electronic density of $n_{\rm e} \approx 10^{10}$ cm^{-2}, this equation yields $T_{\rm e} \approx 10$ K.

Next, we estimate the lower bound on the Floquet gap and the corresponding strength and intensity of the driving electric field. We estimate the lower bound as

the value of the gap at which it equals the scattering rate, $\Delta_{\rm F} = \tau_{\rm scat}^{-1}$. Below this value, the diagonal ensemble of Floquet states is not a proper fixed point for the steady state distribution[4]. Following Eq. (6) in the main text, the scattering rate can be estimated by $\tau_{\rm scat}^{-1} \approx \Gamma_\ell/n_{\rm e} = \Lambda_{\rm inter} n_{\rm h}$. The tightest bound is obtained at the half-filling, where $n_{\rm e} = n_{\rm h} = \sqrt{\kappa}$ [see Eq. (7) in the main text]. Using the definition of κ, we arrive at $\tau_{\rm scat}^{-1} \approx \sqrt{\Gamma_\ell \Lambda_{\rm inter}}$. Using the estimate of $\Lambda_{\rm inter}$ above, we obtain the estimate of the lower bound of the Floquet gap, $\Delta_{\rm min} = [\Gamma_\ell/(\tau_\Lambda^2 \mathcal{A}_{\rm BZ})]^{1/3}$, which yields $\Delta_{\rm min} \approx 0.2$ meV. To estimate the strength of the electric field corresponding to $\Delta_{\rm min}$, we use the equation derived in Ref. 10 for a circularly polarized field:

$$\mathcal{E} \approx \frac{\Delta E_{\rm g}^3}{8k_{\rm R}^2 \lambda_0 (\lambda_0^2 + E_{\rm g}/m_*)}. \quad (125)$$

The value of the electric field strength $\mathcal{E}_{\rm min}$, corresponding to $\Delta = \Delta_{\rm min}$, reads $\mathcal{E}_{\rm min} \approx 4 \times 10^4$ V/m. The field intensity is given by $\mathcal{I} = \frac{1}{2} v_\ell \epsilon_0 \mathcal{E}^2$, where v_ℓ is the speed of light and ϵ_0 is the vacuum permittivity. For $\mathcal{E} = \mathcal{E}_{\rm min}$, we obtain $\mathcal{I}_{\rm min} \approx 2 \times 10^6$ W/m^2.

The upper bound on the Floquet gap is estimated as the value at which heating due to electron-electron interactions become dominant over that arising from radiative recombination, i.e, beyond $\Gamma_{\rm ee} = \Gamma_\ell$. Using the estimate $\Gamma_{\rm ee} \approx \frac{\mathcal{A}_{\rm R}^2 U^2 m_*}{2\pi^4} \left(\frac{V}{\Omega}\right)^2$ [see Supplementary Eq. (62)] and the expression for V (see above), we find $\Delta_{\rm max} \approx \left(\frac{8\pi^4 \Gamma_\ell \lambda_0^2}{\mathcal{A}_{\rm R} m_* U^2}\right)^{\frac{1}{2}}$. For $\tilde{U} = \mathcal{A}_{\rm R} U/\delta E \approx 3$, we obtain $\Delta_{\rm max} \approx 0.2$ eV. The corresponding electric field $\mathcal{E}_{\rm max}$, and intensity $\mathcal{I}_{\rm max}$, read $\mathcal{E}_{\rm max} \approx 4 \times 10^7$ V/m, and $\mathcal{I}_{\rm max} \approx 2 \times 10^{12}$ W/m^2.

* iesin@caltech.edu

[1] L. Lewin, *Polylogarithms and associated functions* (North-Holland, Amsterdam, 1981).
[2] I. Esin, M. S. Rudner, and N. H. Lindner, Sci. Adv. **6**, eaay4922 (2020).
[3] N. W. Ashcroft and N. D. Mermin, *Solid state physics* (Brooks/Cole Cengage Learning, 2003).
[4] K. I. Seetharam, C.-E. Bardyn, N. H. Lindner, M. S. Rudner, and G. Refael, Phys. Rev. X **5**, 041050 (2015).
[5] M. S. Rudner and N. H. Lindner, (2020), arXiv:2003.08252.
[6] K. I. Seetharam, C.-E. Bardyn, N. H. Lindner, M. S. Rudner, and G. Refael, Phys. Rev. B **99**, 014307 (2019).
[7] D. Wood, *The Computation of Polylogarithms*, Tech. Rep. (University of Kent, Computing Laboratory, 1992).
[8] E. Berg, M. S. Rudner, and S. A. Kivelson, Phys. Rev. B **85**, 035116 (2012).
[9] J. Danon, Phys. Rev. B **88**, 075306 (2013).
[10] N. H. Lindner, G. Refael, and V. Galitski, Nat. Phys. **7**, 490 (2011).
